# Genetics of Mayer-Rokitansky-Küster-Hauser (MRKH) syndrome: advancements and implications

**DOI:** 10.3389/fendo.2024.1368990

**Published:** 2024-04-18

**Authors:** Morten Krogh Herlin

**Affiliations:** Department of Clinical Genetics, Aarhus University Hospital, Aarhus N, Denmark

**Keywords:** DNA copy number variations, genetics, genitourinary development, infertility, Mayer-Rokitansky-Küster-Hauser syndrome, MRKH syndrome, MRKHS, Müllerian aplasia

## Abstract

Mayer-Rokitansky-Küster-Hauser (MRKH) syndrome is a congenital anomaly characterized by agenesis/aplasia of the uterus and upper part of the vagina in females with normal external genitalia and a normal female karyotype (46,XX). Patients typically present during adolescence with complaints of primary amenorrhea where the diagnosis is established with significant implications including absolute infertility. Most often cases appear isolated with no family history of MRKH syndrome or related anomalies. However, cumulative reports of familial recurrence suggest genetic factors to be involved. Early candidate gene studies had limited success in their search for genetic causes of MRKH syndrome. More recently, genomic investigations using chromosomal microarray and genome-wide sequencing have been successful in detecting promising genetic variants associated with MRKH syndrome, including 17q12 (*LHX1, HNF1B*) and 16p11.2 (*TBX6*) deletions and sequence variations in *GREB1L* and *PAX8*, pointing towards a heterogeneous etiology with various genes involved. With uterus transplantation as an emerging fertility treatment in MRKH syndrome and increasing evidence for genetic etiologies, the need for genetic counseling concerning the recurrence risk in offspring will likely increase. This review presents the advancements in MRKH syndrome genetics from early familial occurrences and candidate gene searches to current genomic studies. Moreover, the review provides suggestions for future genetic investigations and discusses potential implications for clinical practice.

## 1 Introduction

In mammals, the Müllerian (paramesonephric) ducts give rise to the female reproductive tract, which consists of the Fallopian tubes (oviducts), uterus, cervix, and upper two-thirds of the vagina (1). Abnormalities in Müllerian duct (MD) development in women may result in congenital uterovaginal anomalies of various severity (2, 3).

Mayer-Rokitansky-Küster-Hauser (MRKH) syndrome, also referred to as Müllerian aplasia, is a congenital disorder characterized by agenesis or aplasia of the uterus and upper part of the vagina. The patients are characterized by having a normal female karyotype (46,XX), normal external genitalia, and normal pubertal development of secondary sex characteristics (thelarche and pubarche) (4). MRKH syndrome is typically diagnosed during late adolescence when patients present with primary amenorrhea (5). The estimated birth prevalence of MRKH syndrome is 1 in 5,000 female live births (5, 6) and it is considered the second most common cause of primary amenorrhea (7). MRKH syndrome may present as an isolated anomaly (type I) or in association with extragenital malformations (type II), typically involving the kidneys, skeleton, and heart (4). Upon the diagnosis of MRKH syndrome, patients face life-impacting consequences related to their sexual identity, fear of coital difficulties, and grief of infertility (8–12), and it has been associated with a risk of depressive and anxiety symptoms (13–15). Psychological support and counseling are therefore crucial in patient care (16–18). Non-surgical and surgical treatments of vaginal hypoplasia are available to enable penetrative intercourse with non-surgical dilation considered the first-line choice (18–20). MRKH syndrome causes absolute uterine factor infertility (AUFI) but patients may achieve genetic motherhood through gestational surrogacy (21, 22). In 2014, the first baby was born following pregnancy after uterus transplantation (UTx) in Sweden, offering the first fertility treatment of AUFI achieving both gestational and genetic motherhood (23, 24).

The etiology of MRKH syndrome has long been an unanswered question in medical research, and both genetic and non-genetic factors have been considered. Despite substantial efforts to find explanations for the disorder, our current understanding of the underlying biology remains limited. However, discoveries during recent years do provide evidence for the importance of genetic factors and point towards a heterogeneous etiology with various genes involved in uterovaginal development in humans. This review presents the continuous advancements in our knowledge of genetics in MRKH syndrome, from early candidate gene studies to genome-wide gene discoveries, and provides recommendations for further progress and perspectives on potential clinical implications.

## 2 Embryology of the female reproductive tract

The human genitourinary system (including the kidneys, gonads, and reproductive tracts) develops from the intermediate mesoderm. At 3 weeks post gestation, the Wolffian (mesonephric) ducts, which develop into the male reproductive tracts, form and grow caudally from the primordial kidney (mesonephros) to reach the cloaca. At 5 weeks post gestation, bilateral invaginations of the coelomic epithelium of the urogenital ridges begin to form the MDs which extend caudally, guided by the Wolffian ducts, to reach the urogenital sinus in the midline (Müllerian tubercle). Here, the caudal parts of the two MDs start to fuse to form the uterus and upper vagina starting from week 8. The cranial openings of the invaginations remain and give rise to the fimbrial ends of the oviducts adjacent to the developing ovary (1, 25, 26).

The close relationship between kidney and uterovaginal development is also reflected by the high prevalence (~30%) of kidney malformations in MRKH syndrome (5, 27). Other common extragenital anomalies include the skeleton and heart, which do also develop from the mesoderm, with the paraxial mesoderm forming the axial skeleton (28) and the lateral plate mesoderm forming the heart and appendicular skeleton (29). Therefore, genes involved in the development of mesoderm and its derived structures are relevant candidates in the etiology of MRKH syndrome.

## 3 Evidence for genetic etiologies in MRKH syndrome

Most cases of MRKH syndrome appear isolated with no clear indications of a familial/genetic trait (5, 30). In addition, several reports of discordant monozygotic twin pairs (5, 31–35) and patient-reported outcomes of most surrogate pregnancies also support non-Mendelian causes (36). However, it is important to consider that the disease nature of MRKH syndrome implies absolute infertility, hindering mother-to-offspring inheritance of a genetic cause, which may cause an underestimation of the genetic component of MRKH syndrome from family histories. At this point of knowledge, a single mode of inheritance to cover all cases cannot be determined and monogenic, oligogenic, polygenic, multifactorial, and environmental factors should still be considered. Hypotheses on fetal exposures disturbing uterovaginal development have included thalidomide, diethylstilbestrol, organotins, and phthalates (37–40), but there is no firm evidence for a pathological role in MRKH syndrome.

In 1973, Buchta et al. (41) reported on familial occurrences of various renal malformations following autosomal dominant inheritance and coined the term “*hereditary renal adysplasia*”. One of the patients, who had eight children, was found to have left renal agenesis and a bicorn uterus during surgery for cervical carcinoma *in situ*. Interestingly, 14 years later, John M. Opitz (senior author of the first paper) reported that one daughter of this patient had been diagnosed with MRKH syndrome emphasizing the close link between uterovaginal malformations and renal malformations (42). This association was also described by Schimke & King, who instead proposed the term “*hereditary urogenital adysplasia*” (43). This pattern of both renal and MD anomalies within families, characterized by incomplete penetrance and variable sex-limited expressivity, has been reported several times in the literature (reviewed by Herlin et al. (44)). Taken together this emphasizes the importance of a detailed family history when investigating the genetics of MRKH syndrome.

Recently, the first case of mother-daughter inheritance of MRKH syndrome following gestational surrogacy was reported, when the daughter presented at 14 years old with primary amenorrhea and was diagnosed with type I MRKH syndrome as her mother. A 4 Mb deletion of 2q37.1q37.3 of unknown significance was identified in both patients, but not in the mother’s parents (22). This case exemplifies the potential risk of MRKH syndrome recurrence following assisted reproduction and with the expected increasing availability of UTx as this field moves towards clinical care (24), identifying causal variants and providing genetic counseling regarding recurrence risk and reproductive choices will become more relevant.

## 4 Genetic research in MRKH syndrome

One of the earliest reports describing genetic analysis in the diagnosis of MRKH syndrome is from Georges Andre Hauser in 1961. Hauser and colleagues described that sex-chromatin analysis could aid the differentiation of MRKH syndrome from Turner syndrome and defined MRKH syndrome (at the time termed ‘Mayer-Rokitansky-Küster syndrome’) to include normal female chromosomes (45, 46). Together with earlier anatomical descriptions of Mayer, Rokitansky, and Küster, his work led to the complete definition and its final name (4).

For over 25 years, researchers have searched for genetic alterations to cause uterovaginal agenesis in karyotypically normal women. Early studies investigated a wide range of candidate genes including *GALT* (47–49), *WT1* (50), *HNF1B* (51, 52)*, AMH* (49, 53), *AMHR2* (49, 53), *CFTR* (54), *WNT7A* (55), *HOXA7-13* genes (56–58), *PBX1* (56, 59), *RARG* (60), *RXRA* (60), *CTNNB1* (61), SHOX (62–64), *PAX2* (65), *LAMC1* (66), *DLG1* (66), or *ITIH5* (67). Most of these studies had negative results and provided limited evidence for genetic factors in MRKH syndrome. This included investigations of *AMH* and *AMHR2*, encoding anti-Müllerian hormone and its receptor, respectively, involved in physiological MD regression in males (49, 53). One gene, *Wnt4*, was reported to be involved in female development in mammals with mutant female mice displaying signs of masculinization and absent Müllerian ducts, hence a relevant candidate gene in MRKH syndrome. In 2004, Biason-Lauber et al. identified a *WNT4* variant in an 18-year-old woman with Müllerian agenesis, renal agenesis, and clinical signs of hyperandrogenism. Functional analyses supported the variant pathogenicity and *WNT4* was thereby the first identified gene in uterovaginal agenesis with firm evidence for monogenic causality (discussed further below).

The completion of the Human Genome Project in 2003 (68, 69) together with the concomitant development of genomic technologies including chromosomal microarray (CMA) and massively parallel sequencing (also referred to as Next-Generation Sequencing [NGS]) have allowed for genome-wide (“hypothesis-free”) searches of genetic variation in MRKH syndrome. CMA, including comparative genomic hybridization array and single nucleotide polymorphism (SNP) array, is used to detect copy number variations (CNVs) in the genome. These applications have identified various chromosomal imbalances (deletions/duplications) supporting the identification of candidate genes such as *HNF1B*, *LHX1*, and *TBX6* (**Table 1**) (30, 71, 72, 81, 82). However, recurrent chromosomal imbalances in MRKH syndrome still only apply to a minor fraction of patients (around 10%). Optical genome mapping is a newer cytogenomic method with higher resolution for the detection of both imbalanced and balanced structural variation and has recently been applied in a study by Brakta et al. (73). In more recent years, whole-exome and whole-genome sequencing analyses have been applied for genome-wide detection of genetic variation at the nucleotide level (74, 75, 96–105). Investigations have included both extended familial cases and larger patient cohorts identifying variants of interesting genes, most notably *GREB1L* (99, 101, 102) and *PAX8* (75).

**Table 1:** Recurrent^a^ copy number variations associated with MRKH syndrome.

Locus	Imbalance	CNVs reported	Size range	Region of overlap^b^	Candidate genes	MRKH type	Ref.
16p11.2	Deletion	24	0.5-1.0 Mb	Chr16:29,638,676-30,188,531 (BP4-BP5) (70)	*TBX6*	Type I+II	(30, 71–79)
17q12	Deletion	21	1.4-1.9 Mb	Chr17:36,458,167-37,854,616 (80)	*HNF1B, LHX1*	Type I+II	(30, 71–73, 75, 76, 81–86)
22q11.2	Deletion	7	0.4-5.2 Mb	Various regions involved	Uncertain	Type I+II	(60, 71, 81, 85, 87–90)
	Duplication	4	0.6-3.5 Mb	Various regions involved	Uncertain	Type I	(81, 85, 91, 92)
1q21.1	Deletion	4	0.4-4.6 Mb	Chr1:145,779,056-146,019,795 (82)	*RBM8A*	Type I+II	(30, 82, 85, 93)
2q13q14.1	Deletion	2	9.8-10.8 Mb	Chr2:110,791,355-115,043,578	*PAX8*	Type II	(94, 95)

a Two or more reported CNVs. Non-recurrent candidate variants are listed in **Supplementary T** **able S1**.

b Coordinates in the GRCh38/hg38 reference human genome assembly.

BP, breakpoint; Chr, chromosome; CNV, copy number variation; Mb, megabase.

The vast majority of studies to date have looked into germline genetic variation by analyzing DNA from blood samples. Due to the predominantly sporadic nature of MRKH syndrome and reports of discordant monozygotic twin pairs suggesting non-inherited genetic variation, researchers have also searched for somatic/mosaic gene variation and tissue-specific differential gene methylation/expression patterns in uterine remnants to explain the disorder (35, 98, 106–108).

## 5 Genetic findings in MRKH syndrome and evidence for causality

In this section, the most significant genetic findings to date and the current evidence for causality in MRKH syndrome are discussed. **Table 1** summarizes the recurrent (reported in at least two cases) copy number variations in MRKH syndrome, whereas **Table 2** lists the genes recurrently reported with sequence variants. Non-recurrent (only one case) candidate variants are listed in **Supplementary Table S1**.

**Table 2:** Genes reported with recurrent^a^ sequence variation associated with MRKH syndrome.

Gene	Chromosomal location	Zygosity	Variants reported	Variant type	MRKH type	Other phenotypes/entities associated with gene	Ref.
*GREB1L*	18q11.1-q11.2	Monoallelic	30	Missense (19) Frameshift (5) Splice-site (3) Stop-gain (2) Deletion (1)	Type II (22) Type 1 (8)	CAKUT (renal agenesis/renal hypodysplasia [OMIM #617805]), hearing loss (OMIM #619274), heart malformation, other UVMs	(98, 99, 101, 102, 109, 110)
*TBX6*	16p11.2	Monoallelic/biallelic^b^ (111)	21	Missense (16)^c^ Splice-site (4) Stop-gain (1)	Type II (13) Type I (12)	Scoliosis, spondylocostal dysostosis (OMIM #122600), CAKUT	(72, 75, 103, 105, 111, 112)
*PAX8*	2q14.1	Monoallelic	11	Missense (6) Frameshift (2) Stop-gain (2) Splice-site (1)	Type I (11)	Thyroid hypoplasia/dysgenesis (OMIM #218700)	(75, 98)
*SHOX*	Xp22.33	Monoallelic	10	Duplication (8) Missense (2)	Type I (6) Type II (4)	Leri-Weill dyschondrosteosis (OMIM #127300)	(62, 64, 103)
*WNT9B*	17q21.32	Monoallelic/biallelic^b^ (113)	9	Missense (7) Stop-gain (1) Regulatory (1)	Type I (8)	CAKUT, cleft lip/palate, other UVMs	(98, 103, 113, 114)
*WNT4*	1p36.12	Monoallelic	7	Missense (7)	Type I (6) Type II (1)	Müllerian aplasia and hyperandrogenism (OMIM #158330), other UVMs	(103, 115–118)
*LHX1*	17q12	Monoallelic	6	Missense (5) Frameshift (1)	Type I (2) Type II (1) NS (3)	–	(72, 85, 119, 120)
*LRP10*	14q11.2	Monoallelic/biallelic^b^	5	Missense (5)	Type I (3) Type II (1)	–	(74, 103)
*HNF1B*	17q12	Monoallelic	4	In-frame deletion (2) Frameshift (1) Deletion (1)	Type II	Renal cysts and diabetes syndrome/MODY5 (OMIM #137920)	(51, 52, 121)
*LAMC1*	1q25.3	Monoallelic	4	Missense (4)	Type I (3) Type II (1)	–	(103)
*BMP4*	14q22.2	Monoallelic	3	Stop-gain (2) Splice-site (1)	Type I (2) Type II (1)	Microphthalmia (OMIM **#**607932, cleft lift/palate (OMIM #600625)	(75)
*CTNNA3*	10q21.3	Monoallelic	3	Deletion (3)	Type II (2) Type I (1)	Arrhythmogenic right ventricular dysplasia (OMIM **#**615616)	(73)
*ESR1*	6q25.1-q25.2	Monoallelic	3	Missense (2) Regulatory (1)	Type I	Estrogen resistance (OMIM #615363), breast cancer	(122)
*MMP14*	14q11.2	Monoallelic	3	Missense (2) Duplication (1)	Type I (2) Type II (1)	–	(35, 103)
*RARA*	17q21.2	Monoallelic/biallelic^b^	3	Missense (3)	Type I	Acute promyelocytic leukemia	(103)
*BMP7*	20q13.31	Monoallelic	2	Frameshift (1) Splice-site (1)	Type I	–	(75)
*DLG5*	10q22.3	Monoallelic	2	Missense (1) Stop-gain (1)	Type I+II	CAKUT	(104, 123)
*HOXA10*	7p15.2	Monoallelic	2	Missense (1) Frameshift (1)	Type I+II	Other UVM	(75, 103)
*KMT2D*	12q13.12	Monoallelic	2	Missense (2)	Type I+II	Kabuki syndrome (OMIM# 147920)	(104)
*MKKS*	20p12.2	Monoallelic	2	Missense (2)	Type 2	Bardet-Biedl syndrome (OMIM #605231), McKusick-Kaufman syndrome (OMIM #236700)	(74)
*MYCBP2*	13q22.3	Monoallelic	2	Missense (2)	Type I	–	(97)
*PKD1*	16p13.3	Monoallelic	2	Missense (1) Stop-gain (1)	Type I+II	Polycystic kidney disease (OMIM #173900)	(123)
*SPECC1L*	22q11.23	Monoallelic	2	Missense (2)	Type 2	Teebi hypertelorism syndrome (OMIM #145420)	(74)
*TBC1D1*	4p14	Monoallelic	2	Missense (1) Frameshift (1)	Type I+II	CAKUT	(104)

a Two or more reported sequence variations in the gene. Non-recurrent candidate variants are listed in **Supplementary Table S1**.

b Two variants reported in one case. Phasing was not done to determine *trans* or *cis* configuration.

c Including two polymorphisms (rs56098093 and rs201231713).

CAKUT, congenital anomalies of the kidneys and urinary tracts; MODY5, maturity-onset diabetes of the young type 5; NS, not stated; OMIM, Online Mendelian Inheritance in Man; UVM, uterovaginal malformations.

### 5.1 17q12 deletions, *LHX1*, and *HNF1B*

Deletion of 17q12 was first reported in MRKH syndrome by Cheroki et al. in 2008 (81) and to this date, 21 deletions have been reported (**Table 1**) (30, 71–73, 75, 76, 81–86). Ledig et al. reported a 17q12 deletion in one MRKH syndrome patient (85), whose mother and sister were later reported with the same deletion and other uterovaginal anomalies (124). Deletions of 17q12 are typically 1.4 Mb in size. They are highly penetrant with variable expressivity and cause a multisystem disorder which may include kidney disease, neurocognitive impairment, endocrinological disease including *HNF1B*-related maturity-onset diabetes of the young (MODY), genital malformations, liver disease, and other manifestations (80). Two candidate genes for MRKH syndrome, *LHX1* and *HNF1B*, are located at this locus, both of them being involved in MD development.

*Lhx1*, formerly referred to as *Lim1*, encoding the transcription factor LIM homeobox 1, is involved in normal kidney and MD development (125, 126). *Lhx1*-null female mice have normal ovaries but lack their reproductive tract, which results from a disruption of MD elongation and epithelium formation (125–127). Besides deletions, six single nucleotide variants in *LHX1* (**Table 2**) (72, 85, 119, 120) have been reported including one missense variant with functional evidence of decreased transcriptional activity (120). Still, *LHX1* mutational analysis of larger cohorts did not report any variants, suggesting that sequence variants of *LHX1* are no major cause of MRKH syndrome (30, 128).

*HNF1B* has long been a candidate gene for MRKH syndrome since Lindner et al. already in 1999 reported a Norwegian family with MODY type 5 and progressive parenchymal kidney disease following autosomal dominant inheritance caused by a 75 bp in-frame deletion in exon 2 of *HNF1B*. Notably, two of four female variant carriers also had uterovaginal agenesis, supporting MRKH syndrome as part of the *HNF1B* disease spectrum (51). Subsequent studies have reported *HNF1B* variants associated with various uterovaginal malformations (52, 121, 129, 130), including a ~59 kb whole-gene deletion of *HNF1B* found by WES analysis of a 9-year-old girl presenting with precocious puberty. After the genetic result, reverse phenotyping by imaging confirmed uterus agenesis (121). Recently, Thomson et al. (83) investigated the function of *Hnf1b* following conditional ablation in mice, which resulted in a hypoplastic uterus and renal anomalies (including renal agenesis) similar to an MRKH syndrome type II phenotype. They performed single-cell RNA sequencing of the *Hnf1b*-ablated embryonic uterine tissue and found dysregulated gene expression involved in cell proliferation, patterning, and differentiation. This supports *HNF1B* as a causative gene in MRKH syndrome independent of *LHX1* involvement.

### 5.2 16p11.2 deletions and *TBX6*

In 2011, Nik-Zainal et al. (71) reported four cases with recurrent deletions of 16p11.2 and suggested *TBX6*, previously implicated in paraxial mesoderm development (131), as a candidate gene. To date, a large number of cases have been identified with both deletions (30, 71–79) and *TBX6* sequence variants (72, 75, 103, 105, 111, 112). The 16p11.2/*TBX6* locus is also associated with congenital scoliosis (132). The genetics hereof is complex and does not follow typical Mendelian inheritance, requiring one *TBX6*-null allele and a particular hypomorphic *trans* allele, as described in the compound inheritance gene dosage model (132, 133).

Ma et al. reported 16 rare *TBX6* variants enriched in a large MRKH syndrome patient cohort compared to controls. They performed various functional analyses of 13 missense variants and found evidence for loss-of-function in 7 variants, which together do support the role of *TBX6* variants. However, in contrast to null alleles associated with scoliosis, no second risk alleles were reported in MRKH syndrome (105). As of now, no clear biological mechanism for monoallelic *TBX6* variants causing MRKH syndrome has been established, which challenges interpretation and warrants further studies.

### 5.3 22q11 deletions and duplications

Both deletions (60, 71, 81, 85, 87–90) and duplications (81, 85, 91, 92) of 22q11 have been reported in MRKH syndrome patients (**Table 1**). The CNVs vary in size and location with no single common overlapping region, and therefore no particular candidate genes at 22q11 have been identified. Of note, 22q11 duplication has also been associated with other uterovaginal malformations (124). 22q11 deletions are often associated with DiGeorge and velocardiofacial syndromes. Features hereof are partly thought to be caused by *TBX1* haploinsufficiency, however, some of the deletions reported with MRKH syndrome do not include this particular gene. Overall, the evidence for 22q11 imbalances causing MRKH syndrome remains low.

### 5.4 1q21.1 and *RBM8A*

Variable-sized deletions at 1q21.1 have been associated with MRKH syndrome (30, 82, 85, 93). Duplications of 1q21.1 have also been identified in an MRKH syndrome patient (81) and one case with uterus didelphys (82). The overlapping region has been determined to be GRCh38: chr1:145,779,056-146,019,795 (82) with *RBM8A* as the proposed candidate gene in which sequence variants/polymorphisms also have been identified associated with MRKH syndrome (112).

Deletions of this region may also cause thrombocytopenia–absent radius (TAR) syndrome in compound heterozygosity with certain non-coding polymorphisms on the *trans* allele (134). Notably, TAR syndrome has previously been reported in a case with MRKH syndrome (135, 136). The possible causal role of 1q21.1 deletions/*RBM8A* gene variants in MRKH syndrome is, however, still unclear warranting further studies to establish causality.

### 5.5 2q13q14.1 deletions and *PAX8*

Pathogenic variants in *PAX8* are an established monogenic cause of congenital hypothyroidism due to thyroid dysgenesis (CH, OMIM **#**218700), first described in 1998 by Macchia et al. (137). In an experimental study, *Pax8*-deficient female mice were reported with infertility independently of thyroid replacement therapy, and a possible role in MD development was suggested (138).

Years later, Ma et al. reported on the investigation of a 12-year-old girl with global developmental delay, MD agenesis, and hypothyroidism, found to carry a large 10.79 Mb deletion of 2q13q14.2 (94). A partially overlapping 2q12.1q14.1 deletion was reported in another case with CH, atrial septal defect, intellectual disability, and MD agenesis with anterior displacement of the anus (95). The two deletions share an overlapping region of 4.3 Mb spanning *PAX8* as the proposed candidate gene for MRKH syndrome.

Most recently, Chen et al. (75) reported on a mutational burden analysis of 19 candidate genes based on WES data from 442 cases and 941 controls. Among cases, they found enrichment for predicted loss-of-function variants in *PAX*8. When including results from a replication cohort (n=150), a CH cohort (n=5), and missense variants, a total of 11 *PAX8* variants were found associated with MRKH syndrome. In three cases with available parental DNA, paternal inheritance was confirmed, showing a sex-limited expressivity of infertility. Functional analysis of the five missense variants by luciferase reporter assay found evidence for loss-of-function in two variants. Finally, reverse-phenotyping of five female CH cases, revealed uterovaginal aplasia in one individual, emphasizing the pleiotropy of *PAX8*. This confirms MRKH syndrome as a part of the *PAX8* disease spectrum in females, which the authors refer to as CH-MRKHS (75).

### 5.6 GREB1L

In 2017, three unrelated studies identified *GREB1L* variants as a new autosomal dominant cause of congenital anomalies of the kidney and urinary tract (CAKUT, OMIM #617805) (109, 110, 139). Some of the female cases, mainly fetuses affected by bilateral renal agenesis, were also described with uterovaginal malformations supporting a possible link to MD development (109, 110).

In 2019, Herlin et al. (101) reported on the investigation of a three-generational family with four cases of renal agenesis, including two adult female cousins with MRKH syndrome type II with unilateral renal agenesis. Whole-exome sequencing analysis in this family identified a segregating missense variant in *GREB1L*, supporting *GREB1L* variants as a novel monogenic cause of MRKH syndrome associated with incomplete penetrance and sex-limited expressivity and a phenotype mirroring the early descriptions of *hereditary renal/urogenital adysplasia* (41–43, 140, 141). Jacquinet et al. (102) identified *GREB1L* variants in 5 of 63 (7.9%) sporadic MRKH syndrome patients and segregating variants in four multiplex families presenting renal and uterovaginal malformations. Buchert et al. reported a stop-gain variant in a monozygotic twin-pair discordant for MRKH syndrome with the other twin having unilateral renal agenesis (98). Most recently, Jolly et al. performed an unbiased rare variant enrichment analysis based on WES data from a large American-European cohort (n=148), identifying *GREB1L* as the only gene approaching exome-wide significance based on seven detected variants. From a replication cohort of 442 Han Chinese cases, additional variants were found, including in six cases with type I MRKH syndrome. Of note, besides kidney and uterovaginal malformations, *GREB1L* variants have also been associated with inner ear malformations and deafness as well as complex congenital heart disease (142, 143).

*GREB1L* is considered to be involved in retinoic acid signaling, although its protein remains poorly characterized (109). *Greb1l*-knockdown in zebrafish causes abnormal pronephros morphogenesis (110, 139), and injection of wild-type human mRNA has been shown to rescue the phenotype (110). Knock-in mutagenesis of one missense variant in mice has also been shown to cause renal agenesis (139). Homozygous knock-out of *Greb1l* in mice has been shown to cause absence of the kidneys, Wolffian ducts, and Müllerian ducts (109). However, *GREB1L* variants reported in humans are monoallelic and predominantly missense variants (**Table 2**), and the pathogenic mechanism of how these missense variants cause MRKH syndrome is still unknown requiring further functional analysis.

Taken together, the current evidence with 30 reported variants (**Table 2**) including disease-segregating variants in extended pedigrees, epidemiological evidence of rare variant enrichment in larger cohorts, and functional evidence from knock-out mice, suggest *GREB1L* as a major causative gene in MRKH syndrome.

### 5.7 SHOX

Gervasini et al. (62) first reported on the association of partial *SHOX* duplications in MRKH syndrome in 2010. They investigated 30 cases and 53 controls and identified 5 cases with *SHOX* duplications including a sib-pair with MRKH syndrome. No duplications were identified among controls. Guerrier and Morcel (64) reported similar findings with three *SHOX* duplications and one duplication downstream of the gene. In contrast, Sandbacka et al. found no duplications among 101 Finnish cases questioning the role of *SHOX* in MD development (63). More recently, Mikhael et al. reported two missense variants from an investigation of 72 candidate genes in 111 cases (103). The functional role of these duplications (including their insertion sites) and missense variants is unknown and causality has not been established.

### 5.8 WNT9B

*Wnt9b* encodes a protein involved in MD development and *Wnt9b* knock-down in mice causes uterovaginal and renal agenesis. *Wnt9b* is expressed in the Wolffian duct epithelium providing signals guiding MD elongation (144). *WNT9B* has therefore been considered a candidate gene in MRKH syndrome and has been investigated in several studies identifying a total of 9 sequence variants in MRKH syndrome type I, of these 7 missense variants (**Table 2**) (98, 103, 113, 114). One patient has been reported with two variants (114). Other studies found no variants in MRKH syndrome patients (145, 146). *WNT9B* variants have also been reported in other uterovaginal malformations (114, 146). The functional role of these variants remains to be ascertained.

### 5.9 WNT4

As previously described, *WNT4* was the first gene identified as a monogenic cause of MD agenesis in females (OMIM #158330) (115). Since its discovery, a total of seven missense variants have been reported (103, 115–118). A missense variant of *WNT4* has also been reported with another uterovaginal anomaly with renal agenesis (Herlyn-Werner-Wunderlich syndrome) (147).

Importantly, MD agenesis caused by *WNT4* variants is associated with clinical and biochemical hyperandrogenism, representing a phenotype distinct from MRKH syndrome in general as described by Biason-Lauber et al. (116), and is often considered a separate entity. This is also supported by several investigations reporting no variants in larger MRKH syndrome and MD anomaly cohorts (30, 148, 149).

### 5.10 LRP10

*Lrp10* encodes low-density lipoprotein receptor-related protein 10, which has been proposed as a negative regulator of Wnt/β-catenin signaling (150), a pathway involved in MD development (151). In 2015, Rall et al. reported on a SNP-array analysis of CNVs in discordant twin pairs. In one affected twin, a 585 kb duplication at 14q11.2 spanning *LPR10* was identified, not present in the other twin, and this gene was suggested as a candidate gene for MRKH syndrome. Two subsequent studies have reported a total of five missense variants in *LRP10*, hereof two variants in the same patient (74, 103). Functional evidence for these variants is lacking and causality has not been established.

## 6 Discussion

As described, knowledge of genetic variation in MRKH syndrome has increased considerably during recent years enabled by WES/WGS analysis. In the following, thoughts on how to continue the search for genetic causes are presented and potential implications for future clinical care are discussed.

### 6.1 Family history is key

The development of the field shows the importance of detailed family history in the genetic assessment, as it may include important information to suggest an underlying genetic trait or perhaps provide information on the genetic nature of the family’s trait. The early descriptions of hereditary urogenital adysplasia families, reported long before molecular genetic testing became available, is a good example hereof suggesting an autosomal dominant trait with sex-limited expressivity (41–43, 140, 141). In recent years, *GREB1L* variants have been identified as a major cause of MRKH syndrome, particularly in families with a hereditary urogenital adysplasia-like presentations (101, 102).

Family histories should not only include incidents of MRKH syndrome but also associated extragenital malformations and other uterovaginal malformations in family members. The subtle nature of many renal and uterovaginal malformations is, however, important to consider, as these may not yet have been revealed in asymptomatic relatives, requiring radiological imaging. The relevance of other uterovaginal malformations in the genetic assessment of MRKH syndrome patients is highlighted by the many genes reported in both phenotypes, including 17q12 deletion (85, 124), *EMX2* (75, 152), *GREB1L* (102, 109, 110), *HNF1B* (52, 129, 130)*, HOXA10* (58, 75, 153), *SPECC1L* (74, 154, 155), *WNT4* (115, 147), and *WNT9B* (114). This could suggest a spectrum of uterovaginal anomalies with shared causes.

### 6.2 Future genetic studies

Exome and genome sequencing have proven useful in the discovery of new genes during recent years, including *GREB1L* (99, 101, 102) and *PAX8* (75). With the continuously developing field of genomic sequencing technologies and multi-omics analyses, there is reason to believe that additional genetic causes remain to be identified. Recently, the first complete telomere-to-telomere genome reference was published, which will support even better detection of genetic variation in future genetic studies (156). Also, our understanding of the role of non-coding parts in the genome such as topologically associated domains and long non-coding RNAs is increasing (157, 158). Genome sequencing today is typically based on short-read sequencing due to its cost-effectiveness and accuracy. Long-read sequencing has increased in use and provides some advantages in terms of *de novo* assembly, read-mapping, detection of structural variants, phasing of variants, and transcript isoform identification (159). Another technology for improved analysis of structural variation is optical genome mapping, which has been applied by Brakta et al. (73). All along the technical developments, it will still be relevant to revisit sequencing data of unsolved cases, as new knowledge emerges supporting variant interpretation. Sequenced MRKH syndrome cohorts will increase in size, as will available genomic data resources, which will improve rare variant discovery from association analyses.

It is also relevant to look for somatic variation or differential gene expression in affected tissues as previously performed on surgically-removed uterine remnants (35, 98, 106–108). This may also be helpful in functional analyses of candidate variants of unknown significance to provide evidence for pathogenicity. However, it should be considered that organogenesis in the embryo is an accurately orchestrated process with precise spatiotemporal control of gene expression (160). Therefore, gene expression in adult uterine remnants may not be representative of the gene expression occurring during embryonic development, which in that case would require mouse modeling. As the causal effect of many gene variants remains unknown, functional analyses will be important for further progress.

Finally, it will be important to investigate candidate variants in extended pedigrees to understand the mode of inheritance and disease segregation, also considering more complex mechanisms in developmental genomics as proposed in the compound inheritance gene dosage model (161).

### 6.3 Importance of a genetic diagnosis – possible implications for clinical practice

Identifying genetic causes of MRKH syndrome is of immense importance including both academic pursuits towards improving our understanding of genetic factors underlying human uterovaginal development as well as establishing the necessary evidence to provide informed care and counseling for the individual patient and their families in clinical settings. Rare disease patients often undergo lengthy diagnostic odysseys from the first suspicion until the time of (genetic) diagnosis. Although the diagnostics in MRKH syndrome primarily involve finding the gynecological cause for primary amenorrhea, questions of “*why did this happen*?” remain and feelings of guilt and responsibility are prevalent among patients and their parents (11, 12). Identifying the underlying cause of malformation syndromes may reduce the burden for patients and families, even if it does not lead to changes in treatment (162).

Genetic counseling and management following the identification of a gene variant depends on the ‘clinical actionability’ of the variant, which requires both a valid gene-condition association and sufficient evidence for pathogenicity (163). As evidence for genetic causes in MRKH syndrome continues to increase, it may contribute to better disease classification in the future based on molecular causes instead of the current clinical distinction of type I and II. It can also be expected to guide future patient management such as examinations for associated diseases and anomalies specific to the particular gene. Clinically actionable variants may lead to genetic testing of family members at risk, and variant carriers may then be referred to relevant specialists for further examination. Finally, genetic counseling may address the recurrence risk if patients/couples with an identified genetic cause wish to pursue genetic parenthood either through gestational surrogacy or UTx. The possibility of recurrence in offspring was demonstrated in a recent report, describing the first case of mother-to-daughter inheritance following surrogacy (22). As UTx approaches clinical implementation (24) and the evidence for genetic causes increases, questions about offspring recurrence risk and reproductive choices will likely become more prevalent and so will requests for preimplantation genetic testing as part of the *in vitro* fertilization preceding the UTx procedure.

However, in the current state of knowledge, many reported variants associated with MRKH syndrome are still to be considered as variants of uncertain significance, which warrant cautious interpretations and counseling in clinical care.

## 7 Conclusion

In conclusion, recent advancements provide evidence for genetic causes of MRKH syndrome. The combined evidence points towards a heterogeneous etiology with various genes implicated. With the identification of monogenic causes in MRKH syndrome and increasing fertility options allowing couples to pursue genetic parenthood, the need for genetic counseling will likely increase.

## Author contributions

MKH: Conceptualization, Data curation, Formal analysis, Funding acquisition, Writing – original draft, Writing – review & editing.

## References

[1] Habiba M Heyn R Bianchi P Brosens I Benagiano G. The development of the human uterus: morphogenesis to menarche. Hum Reprod Update. (2021) 27:1–26. doi: 10.1093/humupd/dmaa036 33395479

[2] Grimbizis GF Gordts S Di Spiezio Sardo A Brucker S De Angelis C Gergolet M. The ESHRE/ESGE consensus on the classification of female genital tract congenital anomalies. Hum Reprod. (2013) 28:2032–44. doi: 10.1093/humrep/det098 PMC3712660 23771171

[3] Pfeifer SM Attaran M Goldstein J Lindheim SR Petrozza JC Rackow BW. ASRM müllerian anomalies classification 2021. Fertil Steril. (2021) 116:1238–52. doi: 10.1016/j.fertnstert.2021.09.025 34756327

[4] Herlin MK Petersen MB Brännström M. Mayer-Rokitansky-Küster-Hauser (MRKH) syndrome: a comprehensive update. Orphanet J Rare Dis. (2020) 15:214. doi: 10.1186/s13023-020-01491-9 32819397 PMC7439721

[5] Herlin M Bjorn AM Rasmussen M Trolle B Petersen MB. Prevalence and patient characteristics of Mayer-Rokitansky-Kuster-Hauser syndrome: a nationwide registry-based study. Hum Reprod. (2016) 31:2384–90. doi: 10.1093/humrep/dew220 27609979

[6] Aittomaki K Eroila H Kajanoja P. A population-based study of the incidence of Mullerian aplasia in Finland. Fertil Steril. (2001) 76:624–5. doi: 10.1016/s0015-0282(01)01963-x 11570363

[7] Timmreck LS Reindollar RH. Contemporary issues in primary amenorrhea. Obstet Gynecol Clin North Am. (2003) 30:287–302. doi: 10.1016/S0889-8545(03)00027-5 12836721

[8] Ernst ME Sandberg DE Keegan C Quint EH Lossie AC Yashar BM. The lived experience of MRKH: sharing health information with peers. J Pediatr Adolesc Gynecol. (2016) 29:154–8. doi: 10.1016/j.jpag.2015.09.009 26453829

[9] Patterson CJ Crawford R Jahoda A. Exploring the psychological impact of Mayer-Rokitansky-Küster-Hauser syndrome on young women: An interpretative phenomenological analysis. J Health Psychol. (2016) 21:1228–40. doi: 10.1177/1359105314551077 25293965

[10] Tsitoura A Michala L. The sexuality of adolescents and young women with MRKH syndrome: A qualitative study. J Sex Med. (2021) 18:2012–9. doi: 10.1016/j.jsxm.2021.09.006 34649813

[11] Hatim H Zainuddin AA Anizah A Kalok A Daud TIM Ismail A. The missing uterus, the missed diagnosis, and the missing care. Mayer-rokitansky-küster-hauser syndrome in the lives of women in Malaysia. J Pediatr Adolesc Gynecol. (2021) 34:161–7. doi: 10.1016/j.jpag.2020.11.009 33189898

[12] Jensen AH Herlin MK Vogel I Lou S. A life course perspective on Mayer-Rokitansky-Küster-Hauser syndrome: women’s experiences and negotiations of living with an underdeveloped uterus and vagina. Disabil Rehabil. (2023) 46(6):1130–40. doi: 10.1080/09638288.2023.2191014 36987844

[13] Laggari V Diareme S Christogiorgos S Deligeoroglou E Christopoulos P Tsiantis J. Anxiety and depression in adolescents with polycystic ovary syndrome and Mayer-Rokitansky-Küster-Hauser syndrome. J Psychosom Obstet Gynaecol. (2009) 30:83–8. doi: 10.1080/01674820802546204 19533486

[14] Chen N Song S Duan Y Kang J Deng S Pan H. Study on depressive symptoms in patients with Mayer-Rokitansky-Küster-Hauser syndrome: an analysis of 141 cases. Orphanet J Rare Dis. (2020) 15:121. doi: 10.1186/s13023-020-01405-9 32448241 PMC7245919

[15] Song S Chen N Duan Y-P Kang J Deng S Pan H-X. Anxiety symptoms in patients with Mayer-Rokitansky-Küster-Hauser syndrome: a cross-sectional study. Chin Med J (Engl). (2020) 133:388–94. doi: 10.1097/CM9.0000000000000648 PMC7046242 31977552

[16] Weijenborg PT ter Kuile MM. The effect of a group programme on women with the Mayer-Rokitansky-Küster-Hauser syndrome. BJOG. (2000) 107:365–8. doi: 10.1111/j.1471-0528.2000.tb13232.x 10740333

[17] Heller-Boersma JG Schmidt UH Edmonds DK. A randomized controlled trial of a cognitive-behavioural group intervention versus waiting-list control for women with uterovaginal agenesis (Mayer-Rokitansky-Küster-Hauser syndrome: MRKH). Hum Reprod. (2007) 22:2296–301. doi: 10.1093/humrep/dem167 17584749

[18] ACOG. ACOG committee opinion no. 728: müllerian agenesis: diagnosis, management, and treatment. Obstet Gynecol. (2018) 131:e35–42. doi: 10.1097/AOG.0000000000002458 29266078

[19] Herlin M Bay Bjørn A-M Jørgensen LK Trolle B Petersen MB. Treatment of vaginal agenesis in Mayer-Rokitansky-Küster-Hauser syndrome in Denmark: a nationwide comparative study of anatomical outcome and complications. Fertil Steril. (2018) 110(4):746–53. doi: 10.1016/j.fertnstert.2018.05.015 30196972

[20] Cheikhelard A Bidet M Baptiste A Viaud M Fagot C Khen-Dunlop N. Surgery is not superior to dilation for the management of vaginal agenesis in Mayer-Rokitansky-Küster-Hauser syndrome: a multicenter comparative observational study in 131 patients. Am J Obstet Gynecol. (2018) 219:281.e1–9. doi: 10.1016/j.ajog.2018.07.015 30036500

[21] Friedler S Grin L Liberti G Saar-Ryss B Rabinson Y Meltzer S. The reproductive potential of patients with Mayer-Rokitansky-Küster-Hauser syndrome using gestational surrogacy: a systematic review. Reprod BioMed Online. (2016) 32:54–61. doi: 10.1016/j.rbmo.2015.09.006 26626805

[22] Daum H Kremer E Frumkin A Meiner V Diamant H Harel I. A case report of familial mayer-rokitansky-küster-hauser syndrome as part of the phenotypic spectrum of the 2q37 deletion. J Pediatr Adolesc Gynecology. (2023) 37(1):95–7. doi: 10.1016/j.jpag.2023.09.006 37734585

[23] Brännström M Johannesson L Bokström H Kvarnström N Mölne J Dahm-Kähler P. Livebirth after uterus transplantation. Lancet (London England). (2015) 385:607–16. doi: 10.1016/S0140-6736(14)61728-1 25301505

[24] Brännström M Racowsky C Carbonnel M Wu J Gargiulo A Adashi EY. Uterus transplantation: from research, through human trials and into the future. Hum Reprod Update. (2023) 29:521–44. doi: 10.1093/humupd/dmad012 PMC10477946 37328434

[25] Robboy SJ Kurita T Baskin L Cunha GR. New insights into human female reproductive tract development. Differentiation. (2017) 97:9–22. doi: 10.1016/j.diff.2017.08.002 28918284 PMC5712241

[26] Kobayashi A Behringer RR. Developmental genetics of the female reproductive tract in mammals. Nat Rev Genet. (2003) 4:969–80. doi: 10.1038/nrg1225 14631357

[27] Preibsch H Rall K Wietek BM Brucker SY Staebler A Claussen CD. Clinical value of magnetic resonance imaging in patients with Mayer-Rokitansky-Küster-Hauser (MRKH) syndrome: diagnosis of associated malformations, uterine rudiments and intrauterine endometrium. Eur Radiol. (2014) 24:1621–7. doi: 10.1007/s00330-014-3156-3 24737529

[28] Tani S Chung U Ohba S Hojo H. Understanding paraxial mesoderm development and sclerotome specification for skeletal repair. Exp Mol Med. (2020) 52:1166–77. doi: 10.1038/s12276-020-0482-1 PMC8080658 32788657

[29] Prummel KD Nieuwenhuize S Mosimann C. The lateral plate mesoderm. Development. (2020) 147(12):dev175059. doi: 10.1242/dev.175059 32561665 PMC7328003

[30] Williams LS Demir Eksi D Shen Y Lossie AC Chorich LP Sullivan ME. Genetic analysis of Mayer-Rokitansky-Kuster-Hauser syndrome in a large cohort of families. Fertil Steril. (2017) 108:145–151.e2. doi: 10.1016/j.fertnstert.2017.05.017 28600106 PMC5770980

[31] Lischke JH Curtis CH Lamb EJ. Discordance of vaginal agenesis in monozygotic twins. Obstet Gynecol. (1973) 41:920–4. 4708488

[32] Regenstein AC Berkeley AS. Discordance of müllerian agenesis in monozygotic twins. A case report. J Reprod Med. (1991) 36:396–7. 2061887

[33] Duru UA Laufer MR. Discordance in Mayer-von Rokitansky-Küster-Hauser Syndrome noted in monozygotic twins. J Pediatr Adolesc Gynecol. (2009) 22:e73-5. doi: 10.1016/j.jpag.2008.07.012 19646663

[34] Milsom SR Ogilvie CM Jefferies C Cree L. Discordant Mayer-Rokitansky-Kuster-Hauser (MRKH) syndrome in identical twins - a case report and implications for reproduction in MRKH women. Gynecol Endocrinol Off J Int Soc Gynecol Endocrinol. (2015) 31:684–7. doi: 10.3109/09513590.2015.1032928 26291808

[35] Rall K Eisenbeis S Barresi G Rückner D Walter M Poths S. Mayer-Rokitansky-Küster-Hauser syndrome discordance in monozygotic twins: matrix metalloproteinase 14, low-density lipoprotein receptor-related protein 10, extracellular matrix, and neoangiogenesis genes identified as candidate genes in a tissue-specific. Fertil Steril. (2015) 103:494–502.e3. doi: 10.1016/j.fertnstert.2014.10.053 25492683

[36] Petrozza JC Gray MR Davis AJ Reindollar RH. Congenital absence of the uterus and vagina is not commonly transmitted as a dominant genetic trait: outcomes of surrogate pregnancies. Fertil Steril. (1997) 67:387–9. doi: 10.1016/S0015-0282(97)81927-9 9022619

[37] Hoffmann W Grospietsch G Kuhn W. Thalidomide and female genital malformations. Lancet (London England). (1976) 2:794. England. doi: 10.1016/S0140-6736(76)90618-8 61456

[38] Robboy SJ Taguchi O Cunha GR. Normal development of the human female reproductive tract and alterations resulting from experimental exposure to diethylstilbestrol. Hum Pathol. (1982) 13:190–8. doi: 10.1016/S0046-8177(82)80177-9 7076207

[39] Ema M Miyawaki E Kawashima K. Suppression of uterine decidualization as a cause of implantation failure induced by triphenyltin chloride in rats. Arch Toxicol. (1999) 73:175–9. doi: 10.1007/s002040050603 10401684

[40] Hannas BR Howdeshell KL Furr J Gray LEJ. *In utero* phthalate effects in the female rat: a model for MRKH syndrome. Toxicol Lett. (2013) 223:315–21. doi: 10.1016/j.toxlet.2013.03.021 PMC3971517 23542816

[41] Buchta RM Viseskul C Gilbert EF Sarto GE Opitz JM. Familial bilateral renal agenesis and hereditary renal adysplasia. Z Kinderheilkd. (1973) 115:111–29. doi: 10.1007/BF00440537 4744207

[42] Opitz JM. Vaginal atresia (von Mayer-Rokitansky-Küster or MRK anomaly) in hereditary renal adysplasia (HRA). Am J Med Genet. (1987) 26:873–6. doi: 10.1002/ajmg.1320260414 3591829

[43] Schimke RN King CR. Hereditary urogenital adysplasia. Clin Genet. (1980) 18:417–20. doi: 10.1111/j.1399-0004.1980.tb01786.x 7449179

[44] Herlin M Hojland AT Petersen MB. Familial occurrence of Mayer-Rokitansky-Kuster-Hauser syndrome: a case report and review of the literature. Am J Med Genet A. (2014) 164a:2276–86. doi: 10.1002/ajmg.a.36652 24975471

[45] Hauser GA Schreiner WE. [Mayer-Rokitansky-Kuester syndrome. Rudimentary solid bipartite uterus with solid vagina]. Schweiz Med Wochenschr. (1961) 91:381–4. 13712324

[46] Hauser GA Keller M Koller T Wenner R. [The Rokitansky-Kuester-syndrome. Uterus bipartitus solidus rudimentarius cum vagina solida]. Gynaecologia. (1961) 151:111–2. 13712323

[47] Cramer DW Goldstein DP Fraer C Reichardt JK. Vaginal agenesis (Mayer-Rokitansky-Kuster-Hauser syndrome) associated with the N314D mutation of galactose-1-phosphate uridyl transferase (GALT). Mol Hum Reprod. (1996) 2:145–8. doi: 10.1093/molehr/2.3.145 9238673

[48] Klipstein S Bhagavath B Topipat C Sasur L Reindollar RH Gray MR. The N314D polymorphism of the GALT gene is not associated with congenital absence of the uterus and vagina. Mol Hum Reprod. (2003) 9:171–4. doi: 10.1093/molehr/gag018 12606594

[49] Zenteno JC Carranza-Lira S Kofman-Alfaro S. Molecular analysis of the anti-Müllerian hormone, the anti-Müllerian hormone receptor, and galactose-1-phosphate uridyl transferase genes in patients with the Mayer-Rokitansky-Küster-Hauser syndrome. Arch Gynecol Obstet. (2004) 269:270–3. doi: 10.1007/s00404-002-0456-7 15221321

[50] van Lingen BL Reindollar RH Davis AJ Gray MR. Further evidence that the WT1 gene does not have a role in the development of the derivatives of the müllerian duct. Am J Obstet Gynecol. (1998) 179(3 Pt 1):597–603. doi: 10.1016/s0002-9378(98)70051-1 9757958

[51] Lindner TH Njolstad PR Horikawa Y Bostad L Bell GI Sovik O. A novel syndrome of diabetes mellitus, renal dysfunction and genital malformation associated with a partial deletion of the pseudo-POU domain of hepatocyte nuclear factor-1beta. Hum Mol Genet. (1999) 8:2001–8. doi: 10.1093/hmg/8.11.2001 10484768

[52] Oram RA Edghill EL Blackman J Taylor MJO Kay T Flanagan SE. Mutations in the hepatocyte nuclear factor-1β (HNF1B) gene are common with combined uterine and renal malformations but are not found with isolated uterine malformations. Am J Obstet Gynecol. (2010) 203:364.e1–5. doi: 10.1016/j.ajog.2010.05.022 20633866

[53] Resendes BL Sohn SH Stelling JR Tineo R Davis AJ Gray MR. Role for anti-Müllerian hormone in congenital absence of the uterus and vagina. Am J Med Genet. (2001) 98:129–36. doi: 10.1002/(ISSN)1096-8628 11223848

[54] Timmreck LS Gray MR Handelin B Allito B Rohlfs E Davis AJ. Analysis of cystic fibrosis transmembrane conductance regulator gene mutations in patients with congenital absence of the uterus and vagina. Am J Med Genet A. (2003) 120A:72–6. doi: 10.1002/ajmg.a.20197 12794695

[55] Timmreck LS Pan HA Reindollar RH Gray MR. WNT7A mutations in patients with Müllerian duct abnormalities. J Pediatr Adolesc Gynecol. (2003) 16:217–21. doi: 10.1016/S1083-3188(03)00124-4 14550385

[56] Burel A Mouchel T Odent S Tiker F Knebelmann B Pellerin I. Role of HOXA7 to HOXA13 and PBX1 genes in various forms of MRKH syndrome (congenital absence of uterus and vagina). J Negat Results Biomed. (2006) 5:4. doi: 10.1186/1477-5751-5-4 16556301 PMC1444933

[57] Lalwani S Wu H Reindollar RH Gray MR. HOXA10 mutations in congenital absence of uterus and vagina. Fertil Steril. (2008) 89:325–30. doi: 10.1016/j.fertnstert.2007.03.033 17482600

[58] Ekici AB Strissel PL Oppelt PG Renner SP Brucker S Beckmann MW. HOXA10 and HOXA13 sequence variations in human female genital malformations including congenital absence of the uterus and vagina. Gene. (2013) 518:267–72. doi: 10.1016/j.gene.2013.01.030 23376215

[59] Ma J Qin Y Liu W Duan H Xia M Chen Z-J. Analysis of PBX1 mutations in 192 Chinese women with Müllerian duct abnormalities. Fertil Steril. (2011) 95:2615–7. doi: 10.1016/j.fertnstert.2011.04.074 21575942

[60] Cheroki C Krepischi-Santos AC Rosenberg C Jehee FS Mingroni-Netto RC Pavanello Filho I. Report of a del22q11 in a patient with Mayer-Rokitansky-Küster-Hauser (MRKH) anomaly and exclusion of WNT-4, RAR-gamma, and RXR-alpha as major genes determining MRKH anomaly in a study of 25 affected women. Am J Med Genet A. (2006) 140:1339–42. doi: 10.1002/ajmg.a.31254 16691591

[61] Drummond JB Rezende CF Peixoto FC Carvalho JS Reis FM De Marco L. Molecular analysis of the beta-catenin gene in patients with the Mayer-Rokitansky-Küster-Hauser syndrome. J Assist Reprod Genet. (2008) 25:511–4. doi: 10.1007/s10815-008-9261-y PMC2593771 18979195

[62] Gervasini C Grati FR Lalatta F Tabano S Gentilin B Colapietro P. SHOX duplications found in some cases with type I Mayer-Rokitansky-Kuster-Hauser syndrome. Genet Med Off J Am Coll Med Genet. (2010) 12:634–40. doi: 10.1097/GIM.0b013e3181ed6185 20847698

[63] Sandbacka M Halttunen M Jokimaa V Aittomäki K Laivuori H. Evaluation of SHOX copy number variations in patients with Müllerian aplasia. Orphanet J Rare Dis. (2011) 6:53. doi: 10.1186/1750-1172-6-53 21806840 PMC3159099

[64] Guerrier D Morcel K. Partial SHOX duplications associated with various cases of congenital uterovaginal aplasia (MRKH syndrome): A tangible evidence but a puzzling mechanism. J Genet Med Gene Ther. (2021) 4:1–8. doi: 10.29328/journal.jgmgt

[65] Wang P Zhao H Sun M Li Y Chen Z-J. PAX2 in 192 Chinese women with Müllerian duct abnormalities: mutation analysis. Reprod BioMed Online. (2012) 25:219–22. doi: 10.1016/j.rbmo.2012.04.010 22683154

[66] Ravel C Bashamboo A Bignon-Topalovic J Siffroi J-P McElreavey K Darai E. Polymorphisms in DLGH1 and LAMC1 in mayer-rokitansky-kuster-hauser syndrome. Reprod BioMed Online. (2012) 24:462–5. doi: 10.1016/j.rbmo.2011.12.008 22377151

[67] Morcel K Watrin T Jaffre F Deschamps S Omilli F Pellerin I. Involvement of ITIH5, a candidate gene for congenital uterovaginal aplasia (Mayer-Rokitansky-Küster-Hauser syndrome), in female genital tract development. Gene Expr. (2012) 15:207–14. doi: 10.3727/105221613x13571653093169 PMC6043837 23539898

[68] Lander ES Linton LM Birren B Nusbaum C Zody MC Baldwin J. Initial sequencing and analysis of the human genome. Nature. (2001) 409:860–921. doi: 10.1038/35057062 11237011

[69] Consortium IHGS. Finishing the euchromatic sequence of the human genome. Nature. (2004) 431:931–45. doi: 10.1038/nature03001 15496913

[70] Taylor CM Smith R Lehman C Mitchel MW Singer K Weaver WC. “16p11.2 recurrent deletion”. In: Adam MP Feldman J Mirzaa GM Pagon RA Wallace SE Bean LJH, editors. GeneReviews® [Internet]. Seattle (WA): University of Washington, Seattle (1993). Available at: https://www.ncbi.nlm.nih.gov/books/NBK11167/. 20301775

[71] Nik-Zainal S Strick R Storer M Huang N Rad R Willatt L. High incidence of recurrent copy number variants in patients with isolated and syndromic Mullerian aplasia. J Med Genet. (2011) 48:197–204. doi: 10.1136/jmg.2010.082412 21278390 PMC3322361

[72] Sandbacka M Laivuori H Freitas E Halttunen M Jokimaa V Morin-Papunen L. TBX6, LHX1 and copy number variations in the complex genetics of Mullerian aplasia. Orphanet J Rare Dis. (2013) 8:125. doi: 10.1186/1750-1172-8-125 23954021 PMC3847609

[73] Brakta S Hawkins ZA Sahajpal N Seman N Kira D Chorich LP. Rare structural variants, aneuploidies, and mosaicism in individuals with Mullerian aplasia detected by optical genome mapping. Hum Genet. (2023) 142:483–94. doi: 10.1007/s00439-023-02522-8 36797380

[74] Backhouse B Hanna C Robevska G van den Bergen J Pelosi E Simons C. Identification of candidate genes for mayer-rokitansky-küster-hauser syndrome using genomic approaches. Sex Dev Genet Mol Biol Evol Endocrinol Embryol Pathol sex Determ Differ. (2019) 13:26–34. doi: 10.1159/000494896 30504698

[75] Chen N Zhao S Jolly A Wang L Pan H Yuan J. Perturbations of genes essential for Müllerian duct and Wölffian duct development in Mayer-Rokitansky-Küster-Hauser syndrome. Am J Hum Genet. (2021) 108:337–45. doi: 10.1016/j.ajhg.2020.12.014 PMC7896104 33434492

[76] Pontecorvi P Bernardini L Capalbo A Ceccarelli S Megiorni F Vescarelli E. Protein-protein interaction network analysis applied to DNA copy number profiling suggests new perspectives on the aetiology of Mayer-Rokitansky-Küster-Hauser syndrome. Sci Rep. (2021) 11:448. doi: 10.1038/s41598-020-79827-5 33432050 PMC7801512

[77] Demir Eksi D Shen Y Erman M Chorich LP Sullivan ME Bilekdemir M. Copy number variation and regions of homozygosity analysis in patients with MÜLLERIAN aplasia. Mol Cytogenet. (2018) 11:13. doi: 10.1186/s13039-018-0359-3 29434669 PMC5797403

[78] Gatti M Tolva G Bergamaschi S Giavoli C Esposito S Marchisio P. Mayer-rokitansky-küster-hauser syndrome and 16p11.2 recurrent microdeletion: A case report and review of the literature. J Pediatr Adolesc Gynecol. (2018) 31:533–5. doi: 10.1016/j.jpag.2018.04.003 29730431

[79] Su K Liu H Ye X Jin H Xie Z Yang C. Recurrent human 16p11.2 microdeletions in type I Mayer-Rokitansky-Küster-Hauser (MRKH) syndrome patients in Chinese Han population. Mol Genet Genomic Med. (2023) 12(1):e2280. doi: 10.1002/mgg3.2280 37789575 PMC10767395

[80] Mitchel MW Moreno-De-Luca D Myers SM Levy RV Turner S Ledbetter DH. “17q12 recurrent deletion syndrome”. In: Adam MP Feldman J Mirzaa GM Pagon RA Wallace SE Bean LJH, editors. GeneReviews® [Internet]. Seattle (WA): University of Washington, Seattle (1993). Available at: https://www.ncbi.nlm.nih.gov/books/NBK401562/. 27929632

[81] Cheroki C Krepischi-Santos AC Szuhai K Brenner V Kim CA Otto PA. Genomic imbalances associated with mullerian aplasia. J Med Genet. (2008) 45:228–32. doi: 10.1136/jmg.2007.051839 18039948

[82] McGowan R Tydeman G Shapiro D Craig T Morrison N Logan S. DNA copy number variations are important in the complex genetic architecture of müllerian disorders. Fertil Steril. (2015) 103:1021–30. doi: 10.1016/j.fertnstert.2015.01.008 25707337

[83] Thomson E Tran M Robevska G Ayers K van der Bergen J Gopalakrishnan Bhaskaran P. Functional genomics analysis identifies loss of HNF1B function as a cause of Mayer-Rokitansky-Küster-Hauser syndrome. Hum Mol Genet. (2023) 32:1032–47. doi: 10.1093/hmg/ddac262 PMC9990990 36282544

[84] Bernardini L Gimelli S Gervasini C Carella M Baban A Frontino G. Recurrent microdeletion at 17q12 as a cause of Mayer-Rokitansky-Kuster-Hauser (MRKH) syndrome: two case reports. Orphanet J Rare Dis. (2009) 4:25. doi: 10.1186/1750-1172-4-25 19889212 PMC2777856

[85] Ledig S Schippert C Strick R Beckmann MW Oppelt PG Wieacker P. Recurrent aberrations identified by array-CGH in patients with Mayer-Rokitansky-Kuster-Hauser syndrome. Fertil Steril. (2011) 95:1589–94. doi: 10.1016/j.fertnstert.2010.07.1062 20797712

[86] Hinkes B Hilgers KF Bolz HJ Goppelt-Struebe M Amann K Nagl S. A complex microdeletion 17q12 phenotype in a patient with recurrent *de novo* membranous nephropathy. BMC Nephrol. (2012) 13:27. doi: 10.1186/1471-2369-13-27 22583611 PMC3412739

[87] Devriendt K Moerman P Van Schoubroeck D Vandenberghe K Fryns JP. Chromosome 22q11 deletion presenting as the Potter sequence. J Med Genet. (1997) 34:423–5. doi: 10.1136/jmg.34.5.423 PMC1050953 9152843

[88] Le Caignec C Boceno M Saugier-Veber P Jacquemont S Joubert M David A. Detection of genomic imbalances by array based comparative genomic hybridisation in fetuses with multiple malformations. J Med Genet. (2005) 42:121–8. doi: 10.1136/jmg.2004.025478 PMC1735978 15689449

[89] Morcel K Watrin T Pasquier L Rochard L Le Caignec C Dubourg C. Utero-vaginal aplasia (Mayer-Rokitansky-Kuster-Hauser syndrome) associated with deletions in known DiGeorge or DiGeorge-like loci. Orphanet J Rare Dis. (2011) 6:9. doi: 10.1186/1750-1172-6-9 21406098 PMC3072926

[90] Sundaram UT McDonald-McGinn DM Huff D Emanuel BS Zackai EH Driscoll DA. Primary amenorrhea and absent uterus in the 22q11.2 deletion syndrome. Am J Med Genet A. (2007) 143A:2016–8. doi: 10.1002/ajmg.a.31736 PMC2810967 17676598

[91] AlSubaihin A VanderMeulen J Harris K Duck J McCready E. Müllerian agenesis in cat eye syndrome and 22q11 chromosome abnormalities: A case report and literature review. J Pediatr Adolesc Gynecol. (2018) 31:158–61. doi: 10.1016/j.jpag.2017.09.004 28919146

[92] Dell’Edera D Allegretti A Ventura M Mercuri L Mitidieri A Cuscianna G. Mayer-Rokitansky-Küster-Hauser syndrome with 22q11.21 microduplication: a case report. J Med Case Rep. (2021) 15:208. doi: 10.1186/s13256-021-02716-6 33883018 PMC8058992

[93] Miclea D Alkhzouz C Bucerzan S Grigorescu-Sido P Popp RA Pascanu IM. Molecular and cytogenetic analysis of Romanian patients with differences in sex development. Diagnostics (Basel Switzerland). (2021) 11(11):2107. doi: 10.3390/diagnostics11112107 34829455 PMC8620580

[94] Ma D Marion R Punjabi NP Pereira E Samanich J Agarwal C. A *de novo* 10.79 Mb interstitial deletion at 2q13q14.2 involving PAX8 causing hypothyroidism and mullerian agenesis: A novel case report and literature review. Mol Cytogenet. (2014) 7:4–9. doi: 10.1186/s13039-014-0085-4 25484916 PMC4256837

[95] Smol T Ribero-Karrouz W Edery P Gorduza DB Catteau-Jonard S Manouvrier-Hanu S. Mayer-Rokitansky-Künster-Hauser syndrome due to 2q12.1q14.1 deletion: PAX8 the causing gene? Eur J Med Genet. (2020) 63:103812. doi: 10.1016/j.ejmg.2019.103812 31731040

[96] Chen M-J Wei S-Y Yang W-S Wu T-T Li H-Y Ho H-N. Concurrent exome-targeted next-generation sequencing and single nucleotide polymorphism array to identify the causative genetic aberrations of isolated Mayer-Rokitansky-Küster-Hauser syndrome. Hum Reprod. (2015) 30:1732–42. doi: 10.1093/humrep/dev095 25924657

[97] Takahashi K Hayano T Sugimoto R Kashiwagi H Shinoda M Nishijima Y. Exome and copy number variation analyses of Mayer-Rokitansky-Küster- Hauser syndrome. Hum Genome variation. (2018) 5:27. England. doi: 10.1038/s41439-018-0028-4 PMC6160444 30302266

[98] Buchert R Schenk E Hentrich T Weber N Rall K Sturm M. Genome sequencing and transcriptome profiling in twins discordant for mayer-rokitansky-küster-hauser syndrome. J Clin Med. (2022) 11(19):5598. doi: 10.1101/2022.06.01.22275812 36233463 PMC9573672

[99] Jolly A Du H Borel C Chen N Zhao S Grochowski CM. Rare variant enrichment analysis supports GREB1L as a contributory driver gene in the etiology of Mayer-Rokitansky-Küster-Hauser syndrome. HGG Adv. (2023) 4:100188. doi: 10.1016/j.xhgg.2023.100188 37124138 PMC10130500

[100] Pan H-X Luo G-N Wan S-Q Qin C-L Tang J Zhang M. Detection of *de novo* genetic variants in Mayer-Rokitansky-Küster-Hauser syndrome by whole genome sequencing. Eur J Obstet Gynecol Reprod Biol X. (2019) 4:100089. doi: 10.1016/j.eurox.2019.100089 31517310 PMC6728744

[101] Herlin MK Le VQ Højland AT Ernst A Okkels H Petersen AC. Whole-exome sequencing identifies a GREB1L variant in a three-generation family with Müllerian and renal agenesis: A novel candidate gene in Mayer-Rokitansky-Küster-Hauser (MRKH) syndrome. A case report. Hum Reprod. (2019) 34:1838–46. doi: 10.1093/humrep/dez126 31424080

[102] Jacquinet A Boujemla B Fasquelle C Thiry J Josse C Lumaka A. GREB1L variants in familial and sporadic hereditary urogenital adysplasia and Mayer-Rokitansky-Kuster-Hauser syndrome. Clin Genet. (2020) 98:126–37. doi: 10.1111/cge.13769 32378186

[103] Mikhael S Dugar S Morton M Chorich LP Tam KB Lossie AC. Genetics of agenesis/hypoplasia of the uterus and vagina: narrowing down the number of candidate genes for Mayer-Rokitansky-Küster-Hauser Syndrome. Hum Genet. (2021) 140:667–80. doi: 10.1007/s00439-020-02239-y PMC9211441 33469725

[104] Chu C Li L Li S Zhou Q Zheng P Zhang Y-D. Variants in genes related to development of the urinary system are associated with Mayer-Rokitansky-Küster-Hauser syndrome. Hum Genomics. (2022) 16:10. doi: 10.1186/s40246-022-00385-0 35361250 PMC8969342

[105] Ma C Chen N Jolly A Zhao S Coban-Akdemir Z Tian W. Functional characteristics of a broad spectrum of TBX6 variants in Mayer-Rokitansky-Küster-Hauser syndrome. Genet Med Off J Am Coll Med Genet. (2022) 24:2262–73. doi: 10.1016/j.gim.2022.08.012 36112137

[106] Rall K Barresi G Walter M Poths S Haebig K Schaeferhoff K. A combination of transcriptome and methylation analyses reveals embryologically-relevant candidate genes in MRKH patients. Orphanet J Rare Dis. (2011) 6:32. doi: 10.1186/1750-1172-6-32 21619687 PMC3123171

[107] Hentrich T Koch A Weber N Kilzheimer A Maia A Burkhardt S. The endometrial transcription landscape of MRKH syndrome. Front Cell Dev Biol. (2020) 8:572281. doi: 10.3389/fcell.2020.572281 33072755 PMC7542331

[108] Brucker SY Hentrich T Schulze-Hentrich JM Pietzsch M Wajngarten N Singh AR. Endometrial organoids derived from Mayer-Rokitansky-Küster-Hauser syndrome patients provide insights into disease-causing pathways. Dis Model Mech. (2022) 15(5):dmm049379. doi: 10.1242/dmm.049379 35394036 PMC9118093

[109] De Tomasi L David P Humbert C Silbermann F Arrondel C Tores F. Mutations in GREB1L cause bilateral kidney agenesis in humans and mice. Am J Hum Genet. (2017) 101:803–14. doi: 10.1016/j.ajhg.2017.09.026 PMC5673669 29100091

[110] Sanna-Cherchi S Khan K Westland R Krithivasan P Fievet L Rasouly HM. Exome-wide association study identifies GREB1L mutations in congenital kidney malformations. Am J Hum Genet. (2017) 101:789–802. doi: 10.1016/j.ajhg.2017.09.018 29100090 PMC5673636

[111] Tewes A-C Hucke J Römer T Kapczuk K Schippert C Hillemanns P. Sequence variants in TBX6 are associated with disorders of the müllerian ducts: an update. Sex Dev Genet Mol Biol Evol Endocrinol Embryol Pathol sex Determ Differ. (2019) 13:35–40. doi: 10.1159/000496819 30739119

[112] Tewes AC Rall KK Romer T Hucke J Kapczuk K Brucker S. Variations in RBM8A and TBX6 are associated with disorders of the mullerian ducts. Fertil Steril. (2015) 103:1313–8. doi: 10.1016/j.fertnstert.2015.02.014 25813282

[113] Wang M Li Y Ma W Li H He F Pu D. Analysis of WNT9B mutations in Chinese women with Mayer-Rokitansky-Küster-Hauser syndrome. Reprod BioMed Online. (2014) 28:80–5. doi: 10.1016/j.rbmo.2013.09.022 24268733

[114] Waschk DE Tewes AC Romer T Hucke J Kapczuk K Schippert C. Mutations in WNT9B are associated with Mayer-Rokitansky-Kuster-Hauser syndrome. Clin Genet. (2016) 89(5):590–6. doi: 10.1111/cge.12701 26610373

[115] Biason-Lauber A Konrad D Navratil F Schoenle EJ. A WNT4 mutation associated with Mullerian-duct regression and virilization in a 46,XX woman. N Engl J Med. (2004) 351:792–8. doi: 10.1056/NEJMoa040533 15317892

[116] Biason-Lauber A De Filippo G Konrad D Scarano G Nazzaro A Schoenle EJ. WNT4 deficiency–a clinical phenotype distinct from the classic Mayer-Rokitansky-Kuster-Hauser syndrome: a case report. Hum Reprod. (2007) 22:224–9. doi: 10.1093/humrep/del360 16959810

[117] Philibert P Biason-Lauber A Rouzier R Pienkowski C Paris F Konrad D. Identification and functional analysis of a new WNT4 gene mutation among 28 adolescent girls with primary amenorrhea and müllerian duct abnormalities: a French collaborative study. J Clin Endocrinol Metab. (2008) 93:895–900. doi: 10.1210/jc.2007-2023 18182450

[118] Philibert P Biason-Lauber A Gueorguieva I Stuckens C Pienkowski C Lebon-Labich B. Molecular analysis of WNT4 gene in four adolescent girls with mullerian duct abnormality and hyperandrogenism (atypical Mayer-Rokitansky-Küster-Hauser syndrome). Fertil Steril. (2011) 95:2683–6. doi: 10.1016/j.fertnstert.2011.01.152 21377155

[119] Ledig S Brucker S Barresi G Schomburg J Rall K Wieacker P. Frame shift mutation of LHX1 is associated with Mayer-Rokitansky-Kuster-Hauser (MRKH) syndrome. Hum Reprod. (2012) 27:2872–5. doi: 10.1093/humrep/des206 22740494

[120] Zhang W Zhou X Liu L Zhu Y Liu C Pan H. Identification and functional analysis of a novel LHX1 mutation associated with congenital absence of the uterus and vagina. Oncotarget. (2017) 8:8785–90. doi: 10.18632/oncotarget.v8i5 PMC5352441 28061432

[121] Ai Z Zhu X Chen H Chen R. Precocious puberty or growth hormone deficiency as initial presentation in Mayer-Rokitansky-kuster-Hauser syndrome: a clinical report of 5 cases. BMC Pediatr. (2022) 22:418. doi: 10.1186/s12887-022-03474-0 35836205 PMC9281080

[122] Brucker SY Frank L Eisenbeis S Henes M Wallwiener D Riess O. Sequence variants in ESR1 and OXTR are associated with Mayer-Rokitansky-Küster-Hauser syndrome. Acta Obstet Gynecol Scand. (2017) 96:1338–46. doi: 10.1111/aogs.13202 28815558

[123] Tian W Chen N Ye Y Ma C Qin C Niu Y. A genotype-first analysis in a cohort of Mullerian anomaly. J Hum Genet. (2022) 67:347–52. doi: 10.1038/s10038-021-00996-w 35022528

[124] Ledig S Tewes AC Hucke J Römer T Kapczuk K Schippert C. Array-comparative genomic hybridization analysis in patients with Müllerian fusion anomalies. Clin Genet. (2018) 93:640–6. doi: 10.1111/cge.13160 29068465

[125] Kobayashi A Shawlot W Kania A Behringer RR. Requirement of Lim1 for female reproductive tract development. Development. (2004) 131:539–49. doi: 10.1242/dev.00951 14695376

[126] Huang C-C Orvis GD Kwan KM Behringer RR. Lhx1 is required in Müllerian duct epithelium for uterine development. Dev Biol. (2014) 389:124–36. doi: 10.1016/j.ydbio.2014.01.025 PMC3988469 24560999

[127] Kobayashi A Kwan K-M Carroll TJ McMahon AP Mendelsohn CL Behringer RR. Distinct and sequential tissue-specific activities of the LIM-class homeobox gene Lim1 for tubular morphogenesis during kidney development. Development. (2005) 132:2809–23. doi: 10.1242/dev.01858 15930111

[128] Xia M Zhao H Qin Y Mu Y Wang J Bian Y. LHX1 mutation screening in 96 patients with müllerian duct abnormalities. Fertil Steril. (2012) 97:682–5. doi: 10.1016/j.fertnstert.2011.12.005 22217964

[129] Edghill EL Bingham C Ellard S Hattersley AT. Mutations in hepatocyte nuclear factor-1beta and their related phenotypes. J Med Genet. (2006) 43:84–90. doi: 10.1136/jmg.2005.032854 15930087 PMC2564507

[130] Haumaitre C Fabre M Cormier S Baumann C Delezoide A-L Cereghini S. Severe pancreas hypoplasia and multicystic renal dysplasia in two human fetuses carrying novel HNF1beta/MODY5 mutations. Hum Mol Genet. (2006) 15:2363–75. doi: 10.1093/hmg/ddl161 16801329

[131] White PH Farkas DR McFadden EE Chapman DL. Defective somite patterning in mouse embryos with reduced levels of Tbx6. Development. (2003) 130:1681–90. doi: 10.1242/dev.00367 12620991

[132] Wu N Ming X Xiao J Wu Z Chen X Shinawi M. TBX6 null variants and a common hypomorphic allele in congenital scoliosis. N Engl J Med. (2015) 372:341–50. doi: 10.1056/NEJMoa1406829 PMC4326244 25564734

[133] Liu J Wu N Yang N Takeda K Chen W Li W. TBX6-associated congenital scoliosis (TACS) as a clinically distinguishable subtype of congenital scoliosis: further evidence supporting the compound inheritance and TBX6 gene dosage model. Genet Med. (2019) 21:1548–58. doi: 10.1038/s41436-018-0377-x PMC6659397 30636772

[134] Albers CA Paul DS Schulze H Freson K Stephens JC Smethurst PA. Compound inheritance of a low-frequency regulatory SNP and a rare null mutation in exon-junction complex subunit RBM8A causes TAR syndrome. Nat Genet. (2012) 44:435–9. doi: 10.1038/ng.1083 PMC3428915 22366785

[135] Griesinger G Dafopoulos K Schultze-Mosgau A Schroder A Felberbaum R Diedrich K. Mayer-Rokitansky-Küster-Hauser syndrome associated with thrombocytopenia-absent radius syndrome. Fertil Steril. (2005) 83:452–4. doi: 10.1016/j.fertnstert.2004.06.077 15705390

[136] Ahmad R Pope S. Association of Mayer-Rokitansky-Küster-Hauser syndrome with Thrombocytopenia Absent Radii syndrome: a rare presentation. Eur J obstetrics gynecology Reprod Biol. (2008) 139:257–8. Ireland. doi: 10.1016/j.ejogrb.2007.01.018 17537565

[137] Macchia PE Lapi P Krude H Pirro MT Missero C Chiovato L. PAX8 mutations associated with congenital hypothyroidism caused by thyroid dysgenesis. Nat Genet. (1998) 19:83–6. doi: 10.1038/ng0598-83 9590296

[138] Mittag J Winterhager E Bauer K Grümmer R. Congenital hypothyroid female pax8-deficient mice are infertile despite thyroid hormone replacement therapy. Endocrinology. (2007) 148:719–25. doi: 10.1210/en.2006-1054 17082261

[139] Brophy PD Rasmussen M Parida M Bonde G Darbro BW Hong X. A gene implicated in activation of retinoic acid receptor targets is a novel renal agenesis gene in humans. Genetics. (2017) 207:215–28. doi: 10.1534/genetics.117.1125 PMC5586373 28739660

[140] Pavanello R de C Eigier A Otto PA. Relationship between Mayer-Rokitansky-Küster (MRK) anomaly and hereditary renal adysplasia (HRA). Am J Med Genet. (1988) 29:845–9. doi: 10.1002/ajmg.1320290414 3400728

[141] Battin J Lacombe D Leng JJ. Familial occurrence of hereditary renal adysplasia with müllerian anomalies. Clin Genet. (1993) 43:23–4. doi: 10.1111/j.1399-0004.1993.tb04420.x 8462192

[142] Schrauwen I Kari E Mattox J Llaci L Smeeton J Naymik M. *De novo* variants in GREB1L are associated with non-syndromic inner ear malformations and deafness. Hum Genet. (2018) 137:459–70. doi: 10.1007/s00439-018-1898-8 PMC6082420 29955957

[143] Zhao E Bomback M Khan A Krishna Murthy S Solowiejczyk D Vora NL. The expanded spectrum of human disease associated with GREB1L likely includes complex congenital heart disease. Prenat Diagn. (2024) 44:343–51. doi: 10.1002/pd.6527 PMC11040453 38285371

[144] Carroll TJ Park J-S Hayashi S Majumdar A McMahon AP. Wnt9b plays a central role in the regulation of mesenchymal to epithelial transitions underlying organogenesis of the mammalian urogenital system. Dev Cell. (2005) 9:283–92. doi: 10.1016/j.devcel.2005.05.016 16054034

[145] Ravel C Lorenço D Dessolle L Mandelbaum J McElreavey K Darai E. Mutational analysis of the WNT gene family in women with Mayer-Rokitansky-Kuster-Hauser syndrome. Fertil Steril. (2009) 91:1604–7. doi: 10.1016/j.fertnstert.2008.12.006 19171330

[146] Tang R Dang Y Qin Y Zou S Li G Wang Y. WNT9B in 542 Chinese women with Müllerian duct abnormalities: mutation analysis. Reprod BioMed Online. (2014) 28:503–7. doi: 10.1016/j.rbmo.2013.11.011 24581601

[147] Li L Chu C Li S Lu D Zheng P Sheng J. Renal agenesis-related genes are associated with Herlyn-Werner-Wunderlich syndrome. Fertil Steril. (2021) 116:1360–9. doi: 10.1016/j.fertnstert.2021.06.033 34311961

[148] Clément-Ziza M Khen N Gonzales J Crétolle-Vastel C Picard J-Y Tullio-Pelet A. Exclusion of WNT4 as a major gene in Rokitansky-Küster-Hauser anomaly. Am J Med Genet Part A. (2005) 137:98–9. doi: 10.1002/ajmg.a.30833 16007613

[149] Chang X Qin Y Xu C Li G Zhao X Chen Z-J. Mutations in WNT4 are not responsible for Müllerian duct abnormalities in Chinese women. Reprod BioMed Online. (2012) 24:630–3. doi: 10.1016/j.rbmo.2012.03.008 22503279

[150] Jeong Y-H Sekiya M Hirata M Ye M Yamagishi A Lee S-M. The low-density lipoprotein receptor-related protein 10 is a negative regulator of the canonical Wnt/β-catenin signaling pathway. Biochem Biophys Res Commun. (2010) 392:495–9. doi: 10.1016/j.bbrc.2010.01.049 20093106

[151] Deutscher E Hung-Chang Yao H. Essential roles of mesenchyme-derived beta-catenin in mouse Müllerian duct morphogenesis. Dev Biol. (2007) 307:227–36. doi: 10.1016/j.ydbio.2007.04.036 PMC2020447 17532316

[152] Liu S Gao X Qin Y Liu W Huang T Ma J. Nonsense mutation of EMX2 is potential causative for uterus didelphysis: first molecular explanation for isolated incomplete müllerian fusion. Fertil Steril. (2015) 103:769–74.e2. doi: 10.1016/j.fertnstert.2014.11.030 25577462

[153] Cheng Z Zhu Y Su D Wang J Cheng L Chen B. A novel mutation of HOXA10 in a Chinese woman with a Mullerian duct anomaly. Hum Reprod. (2011) 26:3197–201. doi: 10.1093/humrep/der290 21900391

[154] Kruszka P Li D Harr MH Wilson NR Swarr D McCormick EM. Mutations in SPECC1L, encoding sperm antigen with calponin homology and coiled-coil domains 1-like, are found in some cases of autosomal dominant Opitz G/BBB syndrome. J Med Genet. (2015) 52:104–10. doi: 10.1136/jmedgenet-2014-102677 PMC4393015 25412741

[155] Bhoj EJ Li D Harr MH Tian L Wang T Zhao Y. Expanding the SPECC1L mutation phenotypic spectrum to include Teebi hypertelorism syndrome. Am J Med Genet A. (2015) 167A:2497–502. doi: 10.1002/ajmg.a.37217 26111080

[156] Nurk S Koren S Rhie A Rautiainen M Bzikadze AV Mikheenko A. The complete sequence of a human genome. Science. (2022) 376:44–53. doi: 10.1126/science.abj6987 35357919 PMC9186530

[157] Beagan JA Phillips-Cremins JE. On the existence and functionality of topologically associating domains. Nat Genet. (2020) 52:8–16. doi: 10.1038/s41588-019-0561-1 31925403 PMC7567612

[158] Mattick JS Amaral PP Carninci P Carpenter S Chang HY Chen L-L. Long non-coding RNAs: definitions, functions, challenges and recommendations. Nat Rev Mol Cell Biol. (2023) 24:430–47. doi: 10.1038/s41580-022-00566-8 PMC10213152 36596869

[159] Amarasinghe SL Su S Dong X Zappia L Ritchie ME Gouil Q. Opportunities and challenges in long-read sequencing data analysis. Genome Biol. (2020) 21:30. doi: 10.1186/s13059-020-1935-5 32033565 PMC7006217

[160] Sampath Kumar A Tian L Bolondi A Hernández AA Stickels R Kretzmer H. Spatiotemporal transcriptomic maps of whole mouse embryos at the onset of organogenesis. Nat Genet. (2023) 55:1176–85. doi: 10.1038/s41588-023-01435-6 PMC10335937 37414952

[161] Lupski JR. Clan genomics: From OMIM phenotypic traits to genes and biology. Am J Med Genet A. (2021) 185:3294–313. doi: 10.1002/ajmg.a.62434 PMC8530976 34405553

[162] Manickam K McClain MR Demmer LA Biswas S Kearney HM Malinowski J. Exome and genome sequencing for pediatric patients with congenital anomalies or intellectual disability: an evidence-based clinical guideline of the American College of Medical Genetics and Genomics (ACMG). Genet Med Off J Am Coll Med Genet. (2021) 23:2029–37. doi: 10.1038/s41436-021-01242-6 34211152

[163] Goddard KAB Lee K Buchanan AH Powell BC Hunter JE. Establishing the medical actionability of genomic variants. Annu Rev Genomics Hum Genet. (2022) 23:173–92. doi: 10.1146/annurev-genom-111021-032401 PMC10184682 35363504

