# Supplementary material for: Genetics of Mayer-Rokitansky-Küster-Hauser (MRKH) syndrome: advancements and implications
Source: Front Endocrinol (Lausanne). 2024 Apr 18;15:1368990. doi: 10.3389/fendo.2024.1368990 (PMC11063329; doi:10.3389/fendo.2024.1368990)
Supplement: Supplementary file 1 [file Table_1.docx]

Supplementary Material

# Supplementary Tables

| **Supplementary Table S1.** Non-recurrent candidate variants reported in MRKH syndrome patients | | | | | | |
| --- | --- | --- | --- | --- | --- | --- |
| Variant type | Gene | Chromosomal location | Size | MRKH type | Other phenotypes/ entities associated with gene | Ref. |
| CNV, deletion | *MSH4* | 1p31 | 70 kb | I | Premature ovarian failure (OMIM #619938) Spermatogenic failure (OMIM #108420) | (1) |
| CNV, deletion | Multiple genes | 2p24.1-p24.3 | 4.6 Mb | II | - | (2) |
| CNV, deletion | Multiple genes | 2q11 | 1.5 Mb | II | - | (3) |
| CNV, deletion | *MALL, NPHP1* | 2q13 | 118 kb | II | Joubert syndrome (OMIM # 609583), nephronophthisis (OMIM # 256100), Senior-Loken syndrome (OMIM # 266900) | (4) |
| CNV, deletion | Multiple genes | 2q37.1q37.3 | 4 Mb | I | Chromosome 2q37 deletion syndrome (OMIM #600430) | (5) |
| CNV, deletion | *FGF12* | 3q29 | 50 kb | II | Developmental and epileptic encephalopathy (OMIM #617166) | (4) |
| CNV, deletion | *SLC7A11* | 4q28.3 | 109 kb | I | - | (4) |
| CNV, deletion | *FSTL5* | 4q32.2 | 341 kb | II | - | (4) |
| CNV, deletion | *VEGFC, NEIL3, AGA* | 4q34.2–34.3 | 1.4 Mb | II | Lymphatic malformation (OMIM #615907) | (6) |
| CNV, deletion | Multiple genes | 4q34-qter | 8 Mb | I | - | (7) |
| CNV, deletion | *GNAT3* | 7q21.11 | 55 kb | I | - | (6) |
| CNV, deletion | NS | 7q31.2 | 1.8 Mb | I | - | (8) |
| CNV, deletion | *ZNF277* | 7q31.1 | 21 kb | I | - | (1) |
| CNV, deletion | *FGFR1* | 8p11.2-p11.1 | 25 kb | I | Pfeiffer syndrome (OMIM #101600), Jackson-Weiss syndrome (OMIM #123150) | (1) |
| CNV, deletion | *MSRA* | 8p23 | 1.2 Mb | II | - | (7) |
| CNV, deletion | *SLC30A8* | 8q24.11 | 35 kb | I | - | (6) |
| CNV, deletion | *ITIH5* | 10p14 | 230 kb | II | - | (7) |
| CNV, deletion | *TMEM72-AS1* | 10q11.21 | 102 kb | I | - | (6) |
| CNV, deletion | Multiple genes | 14q32.33 | 460 kb | II | - | (2) |
| CNV, deletion | *SEMA6D* | 15q21.1 | 280 kb | II | - | (2) |
| CNV, deletion | Multiple genes | 18p | NS | II | 18p deletion syndrome (OMIM #146390) | (9) |
| CNV, deletion | *FAAH2* | Xp11.1 | 119 kb | II | - | (4) |
| CNV, deletion | *KLHL4* | Xq21.31 | 1 Mb | II | - | (10) |
| CNV, duplication | *ST6GALNAC3,MSH4, ASB17* | 1p31 | 263 kb | I | Premature ovarian failure (OMIM #619938) Spermatogenic failure (OMIM #108420) | (11) |
| CNV, duplication | *OR2T2* | 1q44 | 179 kb | II | - | (1) |
| CNV, duplication | *ANTXR1, GFPT1* | 2p14 | 232 kb | I | GAPO syndrome (OMIM # 230740), myasthenia (OMIM #610542) | (4) |
| CNV, duplication | *SRD5A2* | 2p23.1 | 210 kb | I | Pseudovaginal perineoscrotal hypospadias (OMIM #264600) | (2) |
| CNV, duplication | Multiple genes | 2q11.2 | 1.3 Mb | II | - | (2) |
| CNV, duplication | *PLA2R1, ITGB6* and *RBMS1* | 2q24.2 | 500 kb | I | Amelogenesis imperfecta (OMIM #616221) | (12) |
| CNV, duplication | *LARS2* | 3p21.31 | 102 kb | II | Perrault syndrome (OMIM #615300) | (4) |
| CNV, duplication | *IFT57, HHLA2* and *MYH15* | 3q13.12q13.13 | 400 kb | II | Orofaciodigital syndrome (OMIM # 617927) | (12) |
| CNV, duplication | *PDE4D* | 5q11.2 | 94 kb | I | Acrodysostosis (OMIM #614613) |  |
| CNV, duplication | *ZFAND3* | 6p21.2 | 174 kb | I | - | (4) |
| CNV, duplication | *PLEKHG1 MTHFD1L* | 6q25.1 | 422 kb | I | - | (4) |
| CNV, duplication | *FBXO5, MTRF1L, RGS17* | 6q25.2 | 442 kb | I | - | (4) |
| CNV, duplication | Multiple genes | 7p14.3 | 1.8 Mb | I | Anemia (OMIM #266120), Bardet-Biedel syndrome (OMIM #615986) | (4) |
| CNV, duplication | *CSMD1* | 8p23 | 1.9 Mb | II | - | (3) |
| CNV, duplication | *CDKN2B* | 9p21 | 73 kb | II | - | (1) |
| CNV, duplication | *ATP5MK, PDCD11, CALHM2* | 10q24.33 | 56 kb | I | - | (4) |
| CNV, duplication | *ANKS1B, BLTP3B* | 12q23.1 | 162 kb | I | - | (4) |
| CNV, duplication | *ACAD10, ALDH2, C12orf47, MAPKAPK5* | 12q24.12 | 125 kb | II | Neurocardiofaciodigital syndrome (OMIM # 619869) | (4) |
| CNV, duplication | *FOXO1* | 13q14.11 | 116 kb | I | - | (11) |
| CNV, duplication | NS | 15q26.3 | 540 kb | I | - | (8) |
| CNV, duplication | *MYOCD* | 17p11.2 | 15 kb | I | Megabladder (OMIM #618719) | (1) |
| CNV, duplication | *CRLF3, ATAD5, TEFM, ADAP2,*  *RNF135* | 17q11.2 | 320 kb | I | Combined oxidative phosphorylation deficiency (OMIM #620451) | (6) |
| CNV, duplication | *TMC8, SYNGR2, TK1, BIRC5,*  *C17orf99, AFMID, TMEM235* | 17q25.3 | 151 kb | I | Epidermodysplasia verruciformis |  |
| CNV, duplication | *SALL3* | 18q23 | 200 kb | I | - | (2) |
| CNV, duplication | *ZNF673, ZNF674* | Xp11.3 | 238 kb | II | - | (4) |
| CNV, duplication | *ARHGAP6, MSL3* | Xp22.2 | 358 kb | I | Basilicata-Akhtar syndrome (OMIM #301032) | (4) |
| CNV, duplication | *COL4A5, IRS4* | Xq22.3 | 93 kb | I | Alport syndrome (OMIM #301050), congenital hypothyroidism (OMIM #301035) | (4) |
| CNV, duplication | *PRKX* | Xp22.33 | 218 kb | II | - | (6) |
| SNV, stop-gain | *ANKRD11* | 16q24.3 | N/A | I | KBG syndrome (OMIM **#**148050) | (13) |
| SNV, stop-gain | *COL11A1* | 1p21 | N/A | I | Marshall syndrome (OMIM #154780), Stickler syndrome type II (OMIM #604841) | (13) |
| SNV, stop-gain | *EMX2* | 10q26.1 | N/A | I | Schizencephaly (OMIM #269160) | (14) |
| SNV, stop-gain | *LEMD3* | 12q14 | N/A | I | Buschke-Ollendorff syndrome (OMIM #166700) | (13) |
| SNV, stop-gain | *TEK* | 9p21 | N/A | I | Glaucoma (OMIM # 617272), venous malformations (OMIM #600195) | (13) |
| SNV, frameshift | *BMP2* | 20p12 | N/A | II | Short stature, facial dysmorphism, and skeletal anomalies with or without cardiac anomalies (OMIM #617877) | (13) |
| SNV, frameshift | *SON* | 21q22.11 | N/A | I | ZTTK syndrome | (13) |
| SNV, frameshift | *SPTB* | 14q23-q24.2 | N/A | II | Elliptocytosis (OMIM #617948), spherocytosis (OMIM #616649) | (13) |
| SNV, splice-site | *BMPR1B* | 4q22-q24 | N/A | I | Acromesomelic dysplasia (OMIM #609441) and brachydactyly (OMIM #616849 and # 112600) | (13) |
| SNV, missense | *ACTR3B* | 7q36.1-q36.2 | N/A | I | - | (15) |
| SNV, missense | *CLIP1* | 12q24.3 | N/A | II | - | (16) |
| SNV, missense | DOCK4 | 7q31.1 | N/A | II | - | (17) |
| SNV, missense | *GATA3* | 10p15 | N/A | I | Hypoparathyroidism, sensorineural deafness, and renal dysplasia (OMIM #146255) | (16) |
| SNV, missense | *GLI3* | 7p13 | N/A | I | Greig cephalopolysyndactyly syndrome (OMIM #604841), Pallister-Hall syndrome (OMIM #146510), polydactyly (OMIM #174200 and #174700) | (16) |
| SNV, missense | *HIRA* | 22q11.21 | N/A | II | - | (16) |
| SNV, missense | *HOXD3* | 2q31.1 | N/A | I | - | (16) |
| SNV, missense | *LIFR* | 5p13-p12 | N/A | I | Stuve-Wiedemann syndrome (OMIM #601559) | (16) |
| SNV, missense | *NAV3* | 12q21.2 | N/A | I | - | (18) |
| SNV, missense | *PAX2* | 10q24 | N/A | I | Papillorenal syndrome (OMIM #120330 | (19) |
| SNV, missense | *PTPN3* | 9q31.3 | N/A | II | - | (18) |
| SNV, missense | *PIK3CD* | 1p36.2 | N/A | I | Immunodeficiency (OMIM #615513 and 619281) | (20) |
| SNV, missense | *RSPO4* | 20p13 | N/A | I | Anonychia congenita (OMIM # 206800) | (17) |
| SNV, missense | *SLC4A10* | 2q23- q24 | N/A | I | Neurodevelopmental disorder with hypotonia and characteristic brain abnormalities (OMIM #620746) | (20) |
| SNV, missense | *TNK2* | 3q29 | N/A | I | - | (20) |
| Abbreviations: CNV, copy number variation; NS, not stated; SNV, single nucleotide variation. | | | | | | |

# Supplementary References

1. Brakta S, Hawkins ZA, Sahajpal N, Seman N, Kira D, Chorich LP, Kim H-G, Xu H, Phillips JA 3rd, Kolhe R, et al. Rare structural variants, aneuploidies, and mosaicism in individuals with Mullerian aplasia detected by optical genome mapping. *Hum Genet* (2023) 142:483–494. doi: 10.1007/s00439-023-02522-8

2. Nik-Zainal S, Strick R, Storer M, Huang N, Rad R, Willatt L, Fitzgerald T, Martin V, Sandford R, Carter NP, et al. High incidence of recurrent copy number variants in patients with isolated and syndromic Mullerian aplasia. *J Med Genet* (2011) 48:197–204. doi: 10.1136/jmg.2010.082412

3. Williams LS, Demir Eksi D, Shen Y, Lossie AC, Chorich LP, Sullivan ME, Phillips JA 3rd, Erman M, Kim H-G, Alper OM, et al. Genetic analysis of Mayer-Rokitansky-Kuster-Hauser syndrome in a large cohort of families. *Fertil Steril* (2017) 108:145-151.e2. doi: 10.1016/j.fertnstert.2017.05.017

4. Ledig S, Schippert C, Strick R, Beckmann MW, Oppelt PG, Wieacker P. Recurrent aberrations identified by array-CGH in patients with Mayer-Rokitansky-Kuster-Hauser syndrome. *Fertil Steril* (2011) 95:1589–1594. doi: 10.1016/j.fertnstert.2010.07.1062

5. Daum H, Kremer E, Frumkin A, Meiner V, Diamant H, Harel I, Bauman D. A Case Report of Familial Mayer-Rokitansky-Küster-Hauser Syndrome as Part of the Phenotypic Spectrum of the 2q37 Deletion. *J Pediatr Adolesc Gynecol* (2023) doi: 10.1016/j.jpag.2023.09.006

6. Pontecorvi P, Bernardini L, Capalbo A, Ceccarelli S, Megiorni F, Vescarelli E, Bottillo I, Preziosi N, Fabbretti M, Perniola G, et al. Protein-protein interaction network analysis applied to DNA copy number profiling suggests new perspectives on the aetiology of Mayer-Rokitansky-Küster-Hauser syndrome. *Sci Rep* (2021) 11:448. doi: 10.1038/s41598-020-79827-5

7. Morcel K, Watrin T, Pasquier L, Rochard L, Le Caignec C, Dubourg C, Loget P, Paniel BJ, Odent S, David V, et al. Utero-vaginal aplasia (Mayer-Rokitansky-Kuster-Hauser syndrome) associated with deletions in known DiGeorge or DiGeorge-like loci. *Orphanet J Rare Dis* (2011) 6:9. doi: 10.1186/1750-1172-6-9

8. McGowan R, Tydeman G, Shapiro D, Craig T, Morrison N, Logan S, Balen AH, Ahmed SF, Deeny M, Tolmie J, et al. DNA copy number variations are important in the complex genetic architecture of müllerian disorders. *Fertil Steril* (2015) 103:1021-1030.e1. doi: 10.1016/j.fertnstert.2015.01.008

9. Anant M, Raj N, Yadav N, Prasad A, Kumar S, Saxena AK. Two Distinctively Rare Syndromes in a Case of Primary Amenorrhea: 18p Deletion and Mayer-Rokitansky-Kuster-Hauser Syndromes. *J Pediatr Genet* (2020) 9:193–197. doi: 10.1055/s-0039-1700577

10. Cheroki C, Krepischi-Santos AC, Szuhai K, Brenner V, Kim CA, Otto PA, Rosenberg C. Genomic imbalances associated with mullerian aplasia. *J Med Genet* (2008) 45:228–232. doi: 10.1136/jmg.2007.051839

11. Demir Eksi D, Shen Y, Erman M, Chorich LP, Sullivan ME, Bilekdemir M, Yılmaz E, Luleci G, Kim H-G, Alper OM, et al. Copy number variation and regions of homozygosity analysis in patients with MÜLLERIAN aplasia. *Mol Cytogenet* (2018) 11:13. doi: 10.1186/s13039-018-0359-3

12. Thomson E, Tran M, Robevska G, Ayers K, van der Bergen J, Gopalakrishnan Bhaskaran P, Haan E, Cereghini S, Vash-Margita A, Margetts M, et al. Functional genomics analysis identifies loss of HNF1B function as a cause of Mayer-Rokitansky-Küster-Hauser syndrome. *Hum Mol Genet* (2023) 32:1032–1047. doi: 10.1093/hmg/ddac262

13. Tian W, Chen N, Ye Y, Ma C, Qin C, Niu Y, Xiaoxin L, Zhao L, Zhao H, Liang Z, et al. A genotype-first analysis in a cohort of Mullerian anomaly. *J Hum Genet* (2022) 67:347–352. doi: 10.1038/s10038-021-00996-w

14. Chen N, Zhao S, Jolly A, Wang L, Pan H, Yuan J, Chen S, Koch A, Ma C, Tian W, et al. Perturbations of genes essential for Müllerian duct and Wölffian duct development in Mayer-Rokitansky-Küster-Hauser syndrome. *Am J Hum Genet* (2021) 108:337–345. doi: 10.1016/j.ajhg.2020.12.014

15. Buchert R, Schenk E, Hentrich T, Weber N, Rall K, Sturm M, Kohlbacher O, Koch A, Riess O, Brucker SY, et al. Genome Sequencing and Transcriptome Profiling in Twins Discordant for Mayer-Rokitansky-Küster-Hauser Syndrome. *J Clin Med* (2022) 11: doi: 10.3390/jcm11195598

16. Chu C, Li L, Li S, Zhou Q, Zheng P, Zhang Y-D, Duan A-H, Lu D, Wu Y-M. Variants in genes related to development of the urinary system are associated with Mayer-Rokitansky-Küster-Hauser syndrome. *Hum Genomics* (2022) 16:10. doi: 10.1186/s40246-022-00385-0

17. Backhouse B, Hanna C, Robevska G, van den Bergen J, Pelosi E, Simons C, Koopman P, Juniarto AZ, Grover S, Faradz S, et al. Identification of Candidate Genes for Mayer-Rokitansky-Küster-Hauser Syndrome Using Genomic Approaches. *Sex Dev Genet Mol Biol Evol Endocrinol Embryol Pathol sex Determ Differ* (2019) 13:26–34. doi: 10.1159/000494896

18. Takahashi K, Hayano T, Sugimoto R, Kashiwagi H, Shinoda M, Nishijima Y, Suzuki T, Suzuki S, Ohnuki Y, Kondo A, et al. Exome and copy number variation analyses of Mayer-Rokitansky-Küster- Hauser syndrome. *Hum genome Var* (2018) 5:27. doi: 10.1038/s41439-018-0028-4

19. Mikhael S, Dugar S, Morton M, Chorich LP, Tam KB, Lossie AC, Kim H-G, Knight J, Taylor HS, Mukherjee S, et al. Genetics of agenesis/hypoplasia of the uterus and vagina: narrowing down the number of candidate genes for Mayer-Rokitansky-Küster-Hauser Syndrome. *Hum Genet* (2021) 140:667–680. doi: 10.1007/s00439-020-02239-y

20. Pan H-X, Luo G-N, Wan S-Q, Qin C-L, Tang J, Zhang M, Du M, Xu K-K, Shi J-Q. Detection of de novo genetic variants in Mayer-Rokitansky-Küster-Hauser syndrome by whole genome sequencing. *Eur J Obstet Gynecol Reprod Biol X* (2019) 4:100089. doi: 10.1016/j.eurox.2019.100089
